# Domain-guided engineering of a thermoresistant Vip3A toxin for enhanced functional robustness

**DOI:** 10.1038/s41598-026-47865-0

**Published:** 2026-04-21

**Authors:** Thanapon Kunlawatwimon, Florian Bourdeaux, Panadda Boonserm, Sumarin Soonsanga, Julian Luka, Boonhiang Promdonkoy, Ulrich Schwaneberg

**Affiliations:** 1 Institute of Molecular Biosciences, Mahidol University, Salaya, Phuttamonthon, Nakhon Pathom, 73170 Thailand; 2 DWI - Leibniz-Institute for Interactive Materials, 52074 Aachen, Germany; 3 Institute of Biotechnology, RWTH Aachen University, 52074 Aachen, Germany; 4 National Center for Genetic Engineering and Biotechnology, National Science and Technology Development Agency, 113 Pahonyothin Road, Khlong Nueng, Khlong Luang, 12120 Pathum Thani Thailand

**Keywords:** Vegetative insecticidal protein, Vip3A, Thermostability engineering, Rational design, Biopesticide, Biochemistry, Biological techniques, Biotechnology

## Abstract

Vip3A toxins from *Bacillus thuringiensis* are effective insecticidal agents for lepidopteran pest control, but their application is limited by poor thermal stability and loss of activity during storage. In this study, we engineered a thermoresistant Vip3Aa64 variant using a combined rational design and directed-evolution approach. Structural analysis and thermal profiling identified domains IV and V as the primary determinants of instability. Rational design targeting domain V yielded stabilizing substitutions that enhanced interdomain interactions, while error-prone mutagenesis of domain IV coupled with high-throughput nanoscale differential scanning fluorimetry screening identified additional mutations conferring increased thermal resistance. Combining the most effective substitutions and removing a mutation that reduced toxicity produced the variant Vip3A-TR6 (I408E/M755K/N633V/G580E), which exhibited a 5.1 °C increase in melting temperature without compromising insecticidal activity against *Spodoptera exigua*. Vip3A-TR6 displayed enhanced resistance to heat-induced aggregation and retained bioactivity after prolonged storage at 25 °C and 37 °C. Importantly, the variant was efficiently produced and secreted by *B. thuringiensis*. These results demonstrate a robust strategy for improving the robustness of Vip3A toxins and support the development of more durable Vip3A-based biopesticides.

**Supplementary Information:**

The online version contains supplementary material available at 10.1038/s41598-026-47865-0.

## Introduction

Biopesticides are a promising alternative to synthetic pesticides because they are generally biodegradable and highly specific in controlling agricultural pests, which helps preserve biodiversity^1^. In addition, biopesticides are typically safe for humans, and substituting traditional pesticides with them reduces the overall quantity of chemical residues in crops and the environment, leading to healthier food production^2^. The biopesticide market has seen substantial growth and is expected to grow even more in the coming years^3^.

Among biopesticides, toxins derived from *Bacillus thuringiensis* are the most significant biocontrol agents used in agriculture, serving both as active constituents in commercial spray formulations and as the basis for *B. thuringiensis* transgenic crops like maize and cotton^4^. Among the numerous toxins produced by *B. thuringiensis*, including crystal or δ-endotoxins (Cry), cytolytic toxins (Cyt), vegetative insecticidal proteins (Vip), and secreted insecticidal proteins (Sip)^4–7^, the three-domain Cry toxins are the most widely used, as they are active against major lepidopteran and coleopteran pests and form stable crystals during sporulation^8^. However, because Cry toxins are used extensively and continuously against key pests like *Helicoverpa armigera* and *Spodoptera frugiperda*, resistance has emerged^9^.

To address resistance against Cry toxins, it was shown that the combination of multiple biopesticides with different mechanisms, such as Cry and Vip toxins, is a promising strategy^10,11^. The potential of these Vip toxins to combat Cry-resistant pests was shown for Cry toxin-resistant *Helicoverpa*, *Spodoptera*, and *Agrotis* insects^12–16^. Vip transgenic plants, such as Agrisure Viptera^®^ maize (Syngenta global AG), VipCot™ (Vip3A and Cry1Ab, Syngenta global AG), and Bollgard^®^ 3 (Cry1Ac, Cry2Ab and Vip3A, Bayer AG) cotton, demonstrate strong effectiveness against Cry toxin-resistant pests^17–19^. In addition, Vip toxins also work synergistically with Cyt proteins, for example, the combination of Vip3Aa and Cyt2Aa showed increased insecticidal effectiveness against *Chilo suppressalis* and *Spodoptera exigua*^20^.

Unlike most Cry toxins, Vip3A toxins do not form crystals and are secreted during the vegetative phase as a protoxin^21^. This protoxin consists of five distinct domains (I-V) arranged in a pyramid-like tetramer^22,23^. Domain I forms a long α-helical bundle that reorganizes into a needle-like structure during proteolytic activation for membrane insertion. Domains II and III form the central core, stabilizing the tetramer and contributing to receptor interaction. The C-terminal domains IV and V adopt β-sandwich folds resembling carbohydrate-binding modules, likely aiding in attachment to sugar-containing receptors on the insect gut surface^22,23^. However, Vip3Aa64 toxins exhibit limited thermal stability, with reported melting temperatures (*T*m) around 56 °C, indicating that they can partially unfold or lose activity under moderate heat stress. This instability contributes to the loss of insecticidal activity during storage and formulation, limiting their broader agricultural application^24–26^. Previous studies had shown that Vip3A undergoes two thermal transitions, with the first *T*m (around 55 °C) corresponding to the unfolding of solvent-exposed domains IV-V, and the second *T*m (around 75 °C) to the unfolding of the tetramerized domains I-III^24–26^.

Improving the thermal resistance of proteins can be achieved through protein engineering approaches such as rational design or directed evolution. Rational design relies on structural and computational insights to identify substitutions that enhance stability, often by lowering molecular flexibility or strengthening intramolecular interactions such as hydrogen bonds, salt bridges, or hydrophobic packing^27^. Computational tools that predict changes in folding free energy (∆∆G) are frequently used to evaluate the stabilizing effect of individual amino acid substitutions before experimental validation^28–32^. In contrast, directed evolution utilizes the generation of variants with random substitutions or shuffled genes, recombining different genes into a new one, and depends on a robust and high-throughput screening system to identify variants with improved characteristics^33–35^. A common method to introduce random mutations in vitro into the target gene or selected region of a gene is error-prone PCR (epPCR), which is easy to use but has bias when introducing mutations, other less biased methods exist, like SeSaM^36^. Semi-rational methods that combine directed evolution approaches with rational design strategies can reduce the amount of screening required and provide further insights into the protein, for example, “KnowVolution”^37^. Improving the thermal resistance of enzymes through rational design or directed evolution has been extensively documented, and numerous strategies have been developed for this purpose^38^. In contrast, protein engineering efforts targeting toxins, particularly insecticidal proteins, are less common and have predominantly focused on modifying specificity or toxicity, as exemplified by studies on Cry toxins^39^. While the intrinsic crystallinity of Cry toxins may reduce the need for stability improvement, explaining the lack of studies in this direction, Vip3A toxins lack such structural stabilization^40^. Despite the need to stabilize Vip3A toxins, only a few studies address this topic^25,26,40–42^. Most reports discussing stability and the few reported engineering approaches are based on creating chimeras^43,44^, highlighting the need for targeted engineering efforts in this direction.

In this study, we used a two-step experimental approach to enhance the thermal stability of Vip3Aa64 while preserving its insecticidal activity. In the first step, we identified the protein’s thermally sensitive domains (domains IV and V) and applied targeted mutagenesis guided by structural analysis and ΔΔG predictions. This was followed by broader random mutagenesis of domain IV using error-prone PCR to explore a wider range of mutations. Combining the two methods yielded a highly stable Vip3Aa64 variant, TR6, which shows significantly increased thermal stability (+ 5.1 °C), paving the way for more durable and effective biopesticide formulations.

## Materials and methods

All chemicals had analytical-reagent grade or higher purity and were purchased from AppliChem (Darmstadt, Germany), Sigma-Aldrich Chemie (Steinheim, Germany), and Carl Roth (Karlsruhe, Germany). All oligonucleotides were acquired from Eurofins Scientific SE (Ebersberg, Germany) in salt-free form. Plasmid extraction and polymerase chain reaction (PCR) product purification were conducted using the NucleoSpin Plasmid Extraction Kit (Macherey-Nagel, Düren, Germany), NEBuilder HiFi DNA Assembly Cloning Kit (New England Biolabs, Ipswich, MA, USA), and the QIAquick PCR Purification Kit (Qiagen, Hilden, Germany), respectively. DNA concentration was measured using a NanoDrop 1000 spectrophotometer (Thermo Scientific, Bremen, Germany).

### *E. coli* strains and recombinant plasmids

Recombinant plasmid pET28b-Vip3Aa64 was as described previously^45^. *E. coli* strains DH5α and BL21-Gold (DE3) were obtained from Agilent Technologies Inc. (Santa Clara, CA, USA). *E. coli* DH5α was used as a cloning host. *E. coli* BL21-Gold (DE3) was used for the expression of Vip3A and the generation of Vip3A mutant libraries.

### Construction of Vip3A domain V variants by site-directed mutagenesis (SDM)

Vip3A variants were constructed by PCR-based site-directed mutagenesis with PCRBIO VeriFi Mix (London, UK), and the mutagenic primers (see **Table ****S01**). The PCR conditions for all SDM were: 95 °C, 1 min, one cycle; 95 °C for 15 s, 61.0 °C for 15 s, 72 °C for 2.40 min, 30 cycles. Following PCR, the products were treated with *Dpn*I (Thermo Fisher Scientific, USA), incubated at 37 ˚C for 2 h. The digested products were examined via agarose gel electrophoresis and purified before being transformed into *E. coli* BL21-Gold (DE3) for expression.

### Directed evolution: generation of domain IV of Vip3A casting error prone PCR (cepPCR) library

Random mutagenesis was performed on the truncated Vip3Aa64 recombinant construct (pET28b-Vip3Aa64(DI-IV) was generated using pET28b-Vip3Aa64 as a template and primers binding outside of DV. The PCR linearized plasmid was transformed into *E. coli* using a Gibson Cloning kit) using error-prone PCR^35^ by varying the concentration of MnCl₂ (25 µM-400 µM) to induce mutations. The cepPCR products were purified and used as megaprimers as described in the MEGAWHOP method^46^. The amplified products were treated with *Dpn*I to degrade the parental methylated plasmid. The resulting mutant plasmids were then electrotransformed into competent *E. coli* BL21-Gold (DE3) cells. Transformants were validated by DNA sequencing to determine mutation load (mutations per kilobase) and amino acid substitutions, enabling the selection of libraries with the desired mutation rates for downstream screening. 0.075 mM MnCl_2_ was selected to produce the Vip3Aa64 (DIV) library.

### Expression of Vip3A in microtiter plates (MTP)

Colonies were picked with sterile toothpicks and placed into a 96-well F-bottom plate containing 140 µL LB media with 50 µg/mL Kanamycin (LB + Kan). The plates were sealed and incubated for 16 h at 37 °C and shaking at 900 rpm. 60 µL of 50% glycerol were added to each well and the plates were sealed and stored at − 80 °C to be used as a master plate. Preculture plates, 96-well flat-bottom MTPs containing 140 µL LB + Kan, were inoculated with a 96-well replicator from the master plate and incubated for 16 h at 37 °C with shaking at 900 rpm. The main culture plates, 96-well V-bottom plates containing 140 µL terrific broth with 50 µg/mL Kanamycin (TB + Kan) per well, were inoculated with 10 µL from the preculture. After 2.5 h incubation at 37 °C and 900 rpm, 15 µL of TB + Kan with 4.4 mM IPTG were added (final IPTG concentration: 0.4 mM). The plates were sealed and incubated for 24 h at 20 °C with shaking at 900 rpm. Cells were harvested by centrifugation at 3,220 rcf, 4 °C, for 30 min. Supernatant was discarded, residual liquid removed by tapping the plate on a paper towel. For cell lysis, lysozyme solution was prepared by dissolving 0.1 mg/mL lysozyme in Tris-HCl buffer (25 mM Tris-HCl pH 8.0). 150 µL of the lysozyme solution were added to each well, and the pellets were resuspended thoroughly. Samples were incubated for 1 h at 37 °C with shaking at 900 rpm. Lysate was clarified by centrifugation at 3,220 rcf and 4 °C for 30 min and directly used for further analysis.

### The flask expression of Vip3A

*E. coli* BL21-Gold (DE3) cells carrying recombinant plasmid pET28b-Vip3A (wild type or mutants) were grown in 5 ml LB + Kan (50 µg/mL) at 37 ℃, 220 rpm for 18 h. After that, the cultures were transferred into 50 ml LB + Kan at 37 ℃, 220 rpm until OD_600_ reached 0.6–0.8. Then, 0.4 mM IPTG was added to induce the expression of the recombinant protein Vip3Aa64. The cultures were continuously grown at 20 ℃ and 220 rpm for 24 h, followed by centrifugation at 3,220 rcf and 4 ℃ for 20 min to harvest the cells^45^. The cell pellets were resuspended in Tris-HCl buffer, and the cells were lysed by ultrasonication. After the centrifugation, the supernatant containing the soluble Vip3A protein was collected for further analysis.

### Purification of Vip3A with Ni-IDA column

The supernatants containing Vip3A wild type or mutants were loaded into Protino Ni-IDA 2000 Packed Columns (Macherey-Nagel, Düren, Germany) that had been pre-equilibrated with Tris-HCl buffer (25 mM Tris-HCl pH 8.0, 250 mM NaCl, 8.6% glycerol). Non-specifically bound proteins were sequentially washed with a washing buffer containing 40 mM and 60 mM imidazole. Then, the recombinant protein was eluted with elution buffer containing 80, 100, and 250 mM imidazole, respectively. The eluted fractions were analyzed by SDS-PAGE. The fractions containing the recombinant protein were concentrated using an Amicon Pro Purification System with a 30 kDa Amicon Ultra-0.5 Device, followed by SDS-PAGE analysis.

### Thermal stability screening using nanoscale differential scanning fluorimetry (NanoDSF)

Melting points were measured with clarified lysates or purified proteins. The samples were loaded into standard-grade capillaries (PR-C002; NanoTemper Technologies) and measured with the Prometheus NT.48 NanoDSF instrument (NanoTemper Technologies). The *T*m was determined by the inflection point of the curve of the fluorescence ratio Em(350 nm)/Em(330 nm) (Ex(280 nm)) measured at gradually increasing temperatures. Measurements were conducted with an excitation power of 50%, starting at 20 °C and increasing to 95 °C at a constant temperature ramp rate of 1.0 °C/min.

### LC_50_ determination (insect bioassays)

The biological assay used the second-instar larvae of *Spodoptera exigua* (provided by the Nuclear Polyhedrosis Virus Production pilot plant at the National Center for Genetic Engineering and Biotechnology, Thailand) in 24-well plates containing artificial diet (1 L consisting of 10 g yeast extract, 1.5 g sorbic acid, 2.5 g ascorbic acid, 20 mL multivitamin stock, 12.6 g agar, and 120 g ground mung bean)^45^. Protein samples were diluted with Tris-HCl buffer (25 mM Tris-HCl, pH 8.0, 250 mM NaCl, 8.6% glycerol) to the required concentration (2000, 1000, 500, 250, 125, 62.5, 31.25, and 15.625 ng/cm^2^) before being applied to the diet surface and allowed to dry prior to placing one larva in each well (12 larvae were tested per replicate, with three replicates per concentration, and three independent experiments were performed). Larval mortality was recorded after seven days at room temperature. The insecticidal effect of each mutant was compared to the wild-type protein (stored at − 80 °C), with Tris-HCl buffer (25 mM Tris-HCl, pH 8.0, 250 mM NaCl, 8.6% glycerol) serving as a negative control. Each bioassay was performed in triplicate, and the LC₅₀ values, indicating the protein concentration required to kill 50% of larvae, were calculated using Probit analysis in IBM SPSS Statistics (version 30). The animal study protocol was approved by the Institute of Molecular Biosciences Animal Care and Use Committee (IMB-ACUC) (COA. NO. IMB-ACUC 2021/019).

### Insecticidal activity after heat treatment

The purified wild-type Vip3A and its mutants (2 µg/cm²) were incubated at 56 °C (*T*m of Vip3A WT) for different time points (1–3 h) and subsequently tested for the biological activity of the heated proteins with *S. exigua.* (12 larvae were tested per replication, and three replications were performed per protein).

### Shelf-life determination of Vip3A and Vip3A-TR6

To assess the shelf-life of the Vip3A protein, a concentration of 2 µg/cm^2^ of Vip3Aa64 and Vip3A-TR6 was stored at 25 and 37 °C for 2 months. The incubated protein was tested with *S. exigua*, and its retained activity was observed weekly as described in *2.7* with the exceptions that no replicates were measured and that 36 larvae were used per condition.

### Construction of Vip3A-TR6 in *B. thuringiensis* 294, Vip3A production, and bioassays

The *vip3Aa64* gene in the native host strain *B. thuringiensis* 294 (Bt294) was modified via CRISPR-Cas9 gene editing strategy to generate the variant Vip3A-TR6, following previously described methods with minor modifications^47^. Briefly, the donor plasmid pBCX-Vip3A-TR6 contained the DNA repair template with the four substitutions (I408E, M755K, N633V, and G580E), while the editing plasmid pLPPR9-Cyt2Pro-Cas9-Svip294 carried a spacer sequence targeting the *vip3Aa64* locus. The plasmids were sequentially introduced into Bt294, and homologous recombination replaced the wild-type gene with the Vip3A-TR6 allele. Following gene editing, both plasmids were cured by culturing transformants in antibiotic-free medium. Correct editing was verified by PCR amplification and DNA sequencing.

For protein production, three individual colonies of Bt294 and Bt294(Vip3A-TR6) were cultivated in Terrific Broth (TB; Difco™) at 30 °C, 200 rpm for 5 days. Cultures were harvested by centrifugation at 12,880 rcf for 30 min at 4 °C, and the supernatants were collected for SDS-PAGE analysis. Vip3A concentrations were estimated by densitometric comparison of full-length Vip3A bands against a bovine serum albumin (BSA).

For heat treatment bioassay, supernatants of Bt294 and Bt294(Vip3A-TR6) were incubated at 50, 55, 60 and 65 °C for 30 min. Samples were tested in diet-overlay bioassays against *S. exigua* at a dose of 0.5 µg/cm^2^ to reflect application-relevant dosing, following the conditions described in Sect. 2.8.

## Results and discussion

### Conceptual design of the protein engineering campaign

**Fig. 1:**
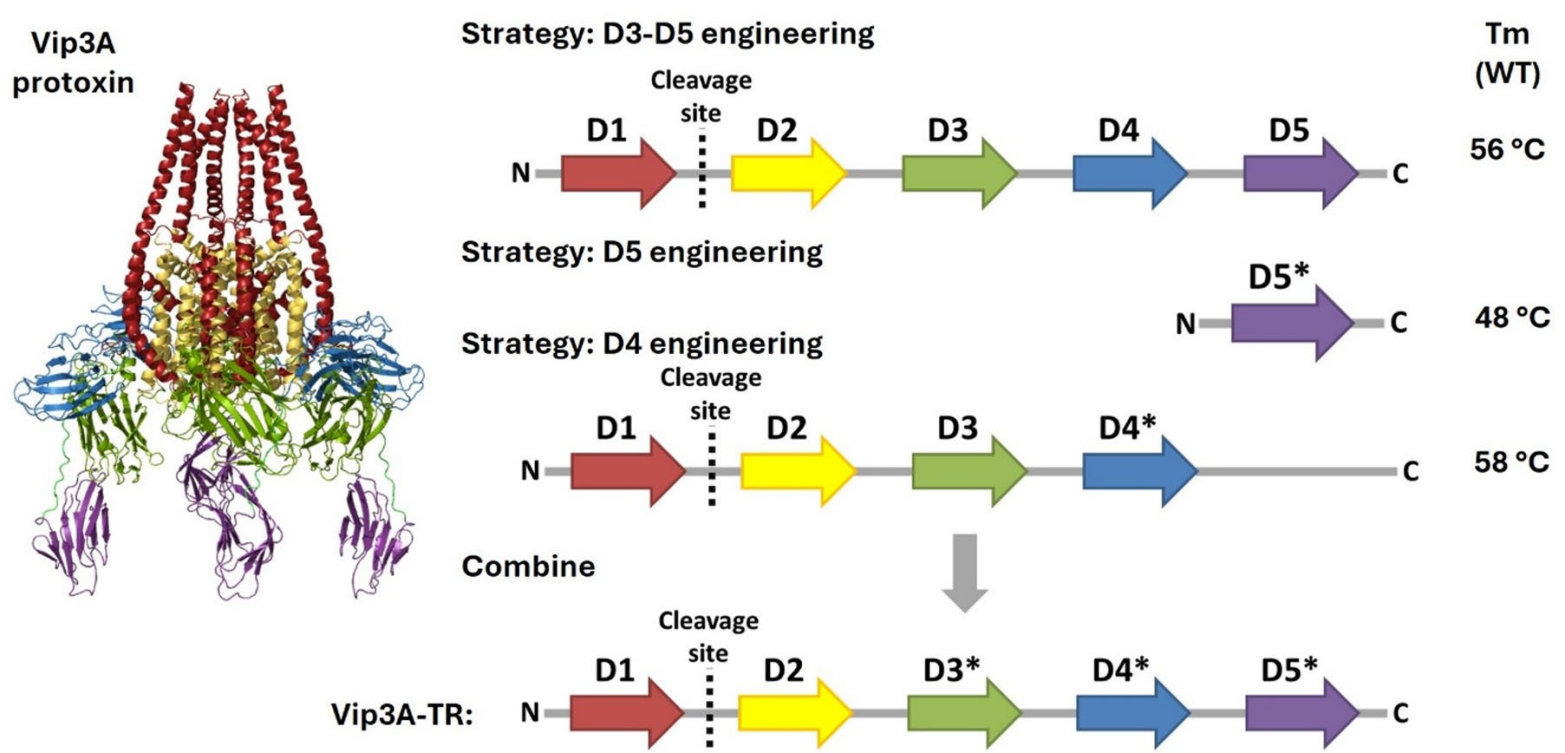
Schematic depiction of the three Vip3Aa64 engineering strategies. Melting points are provided for the WT variants of the tested fragments. Vip3Aa64 protoxin structure was modeled (Swiss-Model^48^ using the structure of Vip3Aa16 (PDB-ID: 6tfj)^22^.

Vip3A toxins exhibit two distinct thermal denaturation events: the first, occurring at approximately 55 °C, corresponds to the unfolding of domains IV and V, while the second, around 75 °C, reflects the dissociation and unfolding of the tetrameric core formed by domains I-III^24–26^. Because domains IV and V represent the least thermally stable regions of Vip3A toxins, they were selected as primary targets for improving overall thermal resistance. Vip3Aa64, a naturally occurring Vip3A variant with higher intrinsic stability than several other Vip3A variants, such as Vip3Aa35^45^, was used as a starting point for the engineering campaign. Initial nanoscale differential scanning fluorimetry (NanoDSF) measurements of Vip3Aa64 (see Fig. S01) indicated that domains IV and V denature cooperatively (only a single denaturing event is observable). Therefore, both domains needed to be improved either simultaneously or in separate proteins. Accordingly, we pursued three engineering strategies (see Fig. 1): (i) stabilization of domain V alone, (ii) stabilization of domain V within the context of the full-length toxin by improving domain III-V interdomain interactions, and (iii) stabilization of domain IV using a truncated construct, Vip3Aa64(DI-DIV), which lacks domain V.

The thermal resistance of all variants generated in this study was assessed using NanoDSF measurements by monitoring changes in the tryptophan fluorescence emission ratio at 330 and 350 nm during a controlled temperature ramp (excitation at 280 nm; see Fig. S01). The generated variants were first analyzed directly in clarified lysates, after which selected variants were purified for detailed analysis. Since only domains IV and V contain tryptophan residues, the assay specifically reports on the unfolding of these two domains (denaturing of domain IV and V coincided with increased light scattering, caused by the aggregation of Vip3Aa64, Fig. S01 c). Initial NanoDSF measurements of isolated domain V, full-length Vip3Aa64, and Vip3Aa64(DI-DIV) confirmed that domain V serves as the starting point for unfolding, as the isolated domain exhibited a low *T*m (47.9 °C) compared to Vip3Aa64 (*T*m = 56.4 °C), and the truncated Vip3Aa64(DI-DIV) construct displayed a higher *T*m (59.1 °C) than Vip3Aa64.

### Protein engineering of domain V

Two strategies were employed focusing on domain V: (i) stabilization of domain V itself and (ii) enhancement of the interaction between domains III and V. Domain V (residues 681–789) is connected to domain IV via a 12-residue linker (residues 669–680) and interacts with domain III through a relatively small interface (see Fig. S02)^22^.

#### Strategy i) improving the thermal resistance of domain V

The strategy of improving the stability of domain V alone did not translate into markedly more stable Vip3Aa64 variants. Based on predictions by the webservers PROSS^49^ and Protposer^50^ and natural substitutions found in Vip3Aa35 multiple substitutions were selected and introduced in domain V (see Table S02). Since the N- (S765) and C-terminus (K789) are in proximity also the introduction of two cysteines to cyclize the domain by formation of a disulfide was evaluated (see Table S02 and Fig. S03). While the single substitution Y776N improved the melting point of domain V by about 2.6 °C and Vip3Aa64 by 0.8 °C (*T*m = 57.2 °C), no other single substitution in domain V was found that improved the thermal resistance of the full-length toxin further. The substitution Y776N occurs naturally in Vip3Aa35 and was supposed to be destabilizing^45^ but surprisingly improved the thermal resistance of Vip3Aa64(DV). NanoDSF measurements with Vip3Aa35, Vip3Aa35(DV), Vip3Aa64 and Vip3Aa64(DV) showed that Vip3Aa64 is more stable than Vip3Aa35, while the domain Vs showed the opposite behavior (*T*m(Vip3Aa64(DV)) > *T*m(Vip3Aa35(DV)); see Table S02). The lower thermal resistance of Vip3Aa35 is likely caused by the altered domain III-V interface (several differences in the sequence regions 716–720 and 755–759). Introduction of cysteines to cyclize domain V, increased the *T*m of the domain by up to 16 °C (variant DV(S765C)-GC, Table S02) but showed only a minor improvement when introduced into Vip3Aa64 (Table S02), indicating that the denaturing of domain V is not main contributor for the denaturing of the whole Vip3Aa64. In addition, preliminary toxicity measurements of these cyclized domain V Vip3Aa64 variants showed near-total loss of activity (mortality tests performed with cell suspensions containing Vip3Aa64 variants producing bacteria; see Table S03). The observed loss of activity may result from reduced conformational flexibility or structural alterations introduced by the disulfide bond. Consequently, although domain V can be substantially stabilized by cyclization in isolation, less structurally disruptive strategies were investigated instead.

#### Strategy ii) improving the interaction between domain III and V

**Fig. 2:**
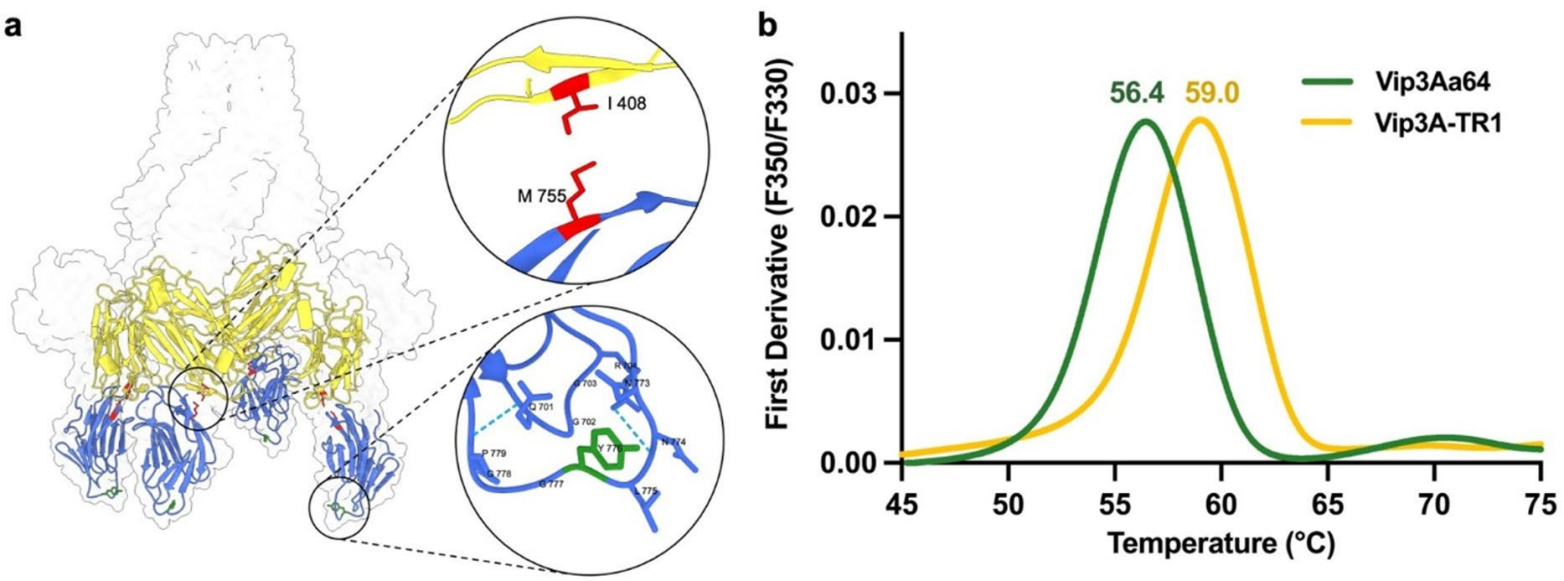
Rational design targeted positions and NanoDSF analysis of a Vip3Aa64 domain V variant. (**a**) Structural model of Vip3Aa64 showing domains III (yellow) and V (blue). Rationally designed mutations I408E, M755K, and Y776N are highlighted at the DIII-DV interface. (**b**) First derivative of the thermal unfolding profiles showing an increase in *T*m from 56.4 °C in the wild type to 59.0 °C in the I408E/M755K/Y776N variant (Vip3Aa64-TR1), indicating improved overall protein stability.

The interface of domain III and V is built up by the domain III regions 408–410, 430–435 and 461–464 the domain V regions 716–720 and 755–759, which consist mostly of loop regions and to some part β-sheets (Fig. S02). All amino acids with side chains pointing toward the interface were selected manually, and potential substitutions were planned to strengthen the interface. Promising pairs of substitutions, introducing a new electrostatic interaction between domain III and V without disrupting present ones, were I408E/M755K (see Fig. 2a) and Y410E/T718K. Both substitution pairs were combined with the substitution Y776N, which stabilizes domain V, and introduced in the full length Vip3Aa64 protein, Vip3Aa64(I408E/M755K/Y776N) and Vip3Aa64(Y410E/T718K/Y776N). Vip3Aa64(I408E/M755K/Y776N) and Vip3Aa64(Y410E/T718K/Y776N) were produced in *E. coli* and the *T*m determined in clarified lysate via NanoDSF. Vip3Aa64(I408E/M755K/Y776N) showed an about 2.5 °C increased *T*m compared to Vip3Aa64 and Vip3Aa64(Y410E/T718K/Y776N) an about 7.3 °C reduced one. Vip3Aa64(I408E/M755K/Y776N), named Vip3Aa64-TR1 (thermoresistant variant 1), was purified and the *T*m determined via NanoDSF (see Fig. 2b), Vip3Aa64-TR1 exhibited a 2.6 °C increased *T*m (59.0 °C) compared to Vip3Aa64 (*T*m = 56.4 °C).

### Protein engineering of domain IV

In contrast to domain V, no clear rational design strategy was identified during the structural analysis, therefore a directed evolution campaign was conducted to stabilize domain IV (strategy (iii)). To measure only the denaturing of domain IV, the truncated construct Vip3Aa6(DI-IV) was used. Vip3Aa6(DI-IV) could be produced similarly to Vip3Aa64 and performed well in lysate and purified form. Random mutations were introduced into the domain IV coding region by casting error prone PCR (cepPCR) using 0.075 mM MnCl_2_. The cepPCR fragment was incorporated into the Vip3Aa6(DI-IV) coding plasmid via MEGAWHOP cloning and transformed into *E. coli* to generate the screening libraries^46^. A mutation rate of 4.2 mutations/kb was determined by sequencing 30 clones, corresponding to about 1.7 mutations per domain IV gene (405 bp). The screening library contained in total 1,936 clones.

Thermal resistance of the Vip3Aa64(DI-IV) variants was measured in clarified lysate via NanoDSF. Vip3Aa6(DI-IV) variants for screening were produced in 96-well MTPs and selected variants were produced in flasks and purified. Enzymatic lysis was performed under the following conditions: 25 mM Tris-HCl (pH 8.0) with 0.1 mg/mL lysozyme at 37 °C and 900 rpm shaking. Lysozyme was used at a low concentration to avoid interference with NanoDSF measurements. The robustness of the screening workflow, including MTP expression, enzymatic lysis, and NanoDSF analysis in clarified lysates, was assessed by measuring 96 replicates of Vip3Aa64(DI-IV) (see Fig. S04). The mean *T*m of 59.1 °C was consistent with values obtained from flask-based expression, and the low coefficient of variation (CV = 0.26%) confirms the reliability and suitability of the screening system.

**Fig. 3:**
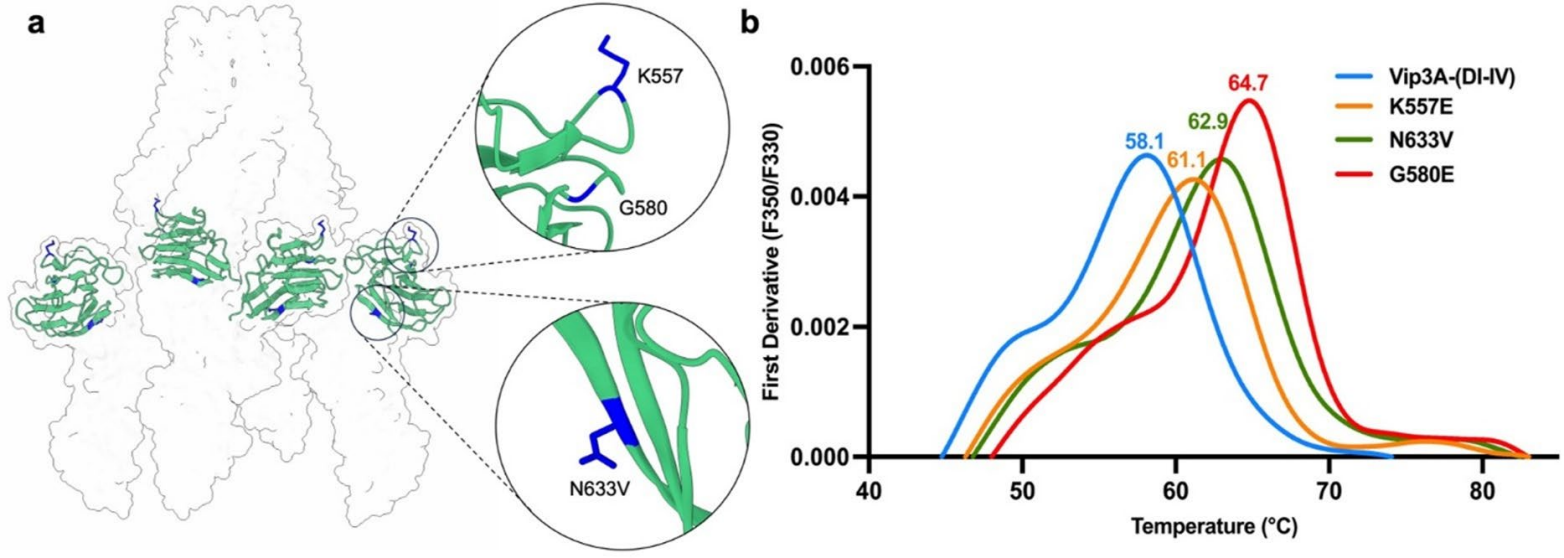
Domain IV positions found during screening and computational predictions and NanoDSF analysis of a Vip3Aa64 domain IV variants. (**a**) Structural model of Vip3Aa64 showing domains IV (green) and the residues K557, G580, and N633 are highlighted. (**b**) First derivative of the thermal unfolding profiles of Vip3Aa64(DI-DIV) variants.

In total, four Vip3Aa64(DI-IV) variants with enhanced thermal resistance (Δ*T*m > + 1 °C) were identified across three positions (see Table S04), whereas most screened variants showed *T*m values comparable to the parental Vip3A(DI-IV). Among the variants, Vip3Aa64(DI-IV(G580E)) displayed the greatest improvement, with a 6.6 °C increase in *T*m (see Fig. 3). Moderate improvements were observed in Vip3Aa64(DI-IV(K557E)) and Vip3Aa64(DI-IV(N633S)), each exhibiting a 3.0 °C increase in *T*m. The introduction of the G580E substitution into Vip3Aa64 resulted in only a modest increase in *T*m (+ 1.0 °C, see Table S05), consistent with the interpretation that dissociation of domain V from domain III is a primary contributor to denaturation by increasing mechanical strain on domain IV via the linker.

In addition to the random mutagenesis potential beneficial substitutions were predicted using FoldX^51^, I-Mutant^52^, and DeepDDG^53^. The criteria were adjusted from the method described in Xu, S. Y. et al., 2024^54^. Predicted candidates were selected from the intersection of these programs. In FoldX, negative values indicate improved stability, whereas positive values in DeepDDG and I-Mutant represent improved stability. Substitutions at each residue with a total energy of < − 10.0 kcal/mol were selected. Substitutions with the highest positive values in both DeepDDG and I-Mutant were chosen for further investigation. Based on manual structural analysis and predicted ΔΔG values, 10 substitutions (see Table S06) were selected and introduced into Vip3A(DI-IV). Two of the selected substitutions were in the same positions as found by the random mutagenesis, K557 and N633. Of the predicted variants, only Vip3Aa64(DI-IV(K557P)) and Vip3Aa64(DI-IV(N633V)), the same positions found in the mutagenesis campaign, showed a notable improvement in thermal resistance compared to Vip3Aa64(DI-IV), increased by + 1.8 °C and + 4.8 °C, respectively (Table S04). The other predicted variants overall had lower *T*m values, indicating that, given the low number of substitutions in the mutagenesis campaign, the structure of domain IV appears sensitive to alterations.

The structural effects of the substitutions at positions K557, G580, and N633 on the stability of Vip3Aa64 remain inconclusive, as molecular dynamics simulations did not reveal significant changes in the structure or dynamics of domain IV (Fig. S05). In contrast, the introduction of a salt bridge between domains III and V (I408E/M755K) had a pronounced effect on the dynamics of domain V. Specifically, the root-mean-square fluctuation (RMSF) of domain V in Vip3A-TR6 was reduced under all simulated conditions (Fig. S05 a-c), and the interdomain distance between domains III and V exhibited decreased fluctuation and remained consistently shorter compared to Vip3Aa64 (Fig. S05 d). One possible explanation for the lack of observable effects in domain IV is that the simulation conditions (300 K and 330 K; 200 ns) were insufficient to capture relevant dynamic changes, particularly given the comparatively rigid nature of domain IV relative to domain V (Fig. S05 a-c). Also structural analysis of the mutation sites did not provide definitive mechanistic insights. Residue G580 is located at the end of a short β-sheet in domain IV and is positioned near K650 (see Fig. S06 a), suggesting that the G580E substitution may introduce stabilizing electrostatic interactions. Residue N633 is located at the interface between domains III and IV (Fig. S06 b). The closest residue from domain III is I367, which, together with V596, N633, and R635 (whose guanidinium group is oriented away from I367), forms a small hydrophobic patch (Fig. S06 b). Residue K557 is located within a highly exposed loop and lacks obvious structural interaction partners (Fig. S06 a).

### Introducing the substitutions into Vip3Aa64

The substitutions found in strategy (iii), K557E, G580E, and N633V, were introduced into Vip3A-TR1 to generate new thermal-resistant variants (see Table 1). All Vip3A-TR variants were produced in flask cultures, purified via His-tag affinity chromatography, and the buffer was exchanged to remove imidazole. The thermal resistance of all variants was determined using NanoDSF.

**Table 1:** *T*m values and LC_50_ against *S. exigua* of purified Vip3A-TR variants. All samples were measured in technical triplicates. Standard deviation of the *T*m values was for all samples below 0.1 °C (for the first derivative of the NanoDSF measurements see Fig. S07).

Vip3A variant	Substitution	Tm (°C)	LC_50_ (ng/cm^2^)^a^
Vip3Aa64	-	56.4 ± 0.1	36 (25–49)
Vip3A-TR1	I408E/M755K/Y776N	59.0 ± 0.1	354 (282–444)
Vip3A-TR2	I408E/M755K/Y776N/K557E	59.3 ± 0.1	-
Vip3A-TR3	I408E/M755K/Y776N/G580E	61.5 ± 0.1	385 (306–487)
Vip3A-TR4	I408E/M755K/Y776N/N633V	60.4 ± 0.1	375 (293–480)
Vip3A-TR5	I408E/M755K/Y776N/N633V/G580E	62.9 ± 0.1	456 (356–591)
Vip3A-TR6	I408E/M755K/N633V/G580E	61.5 ± 0.1	35 (24–48)

^a^95% confidence limits are shown in parentheses from three independent experiments.

The substitutions G580 and N633V both increased the thermal resistances of Vip3A-TR1 by more than 1 °C, while K557E showed only a negligible improvement (+ 0.3 °C) and was not further investigated. The combination of G580E and N633V (Vip3A-TR5) increased the *T*m by 3.9 °C compared to Vip3A-TR1, which is equal to the sum of improvements exhibited by the single substitutions (Vip3A-TR3: +2.5 °C; Vip3A-TR4: +1.4 °C). This synergistic effect is consistent with the proposed distinct stabilization mechanisms: N633V improves domain III-IV interactions, and G580E stabilizes the fold of domain IV.

Insecticidal activity of all Vip3A-TR variants, except Vip3A-TR2, was assessed in larval feeding assays, and LC₅₀ values were determined (Table 1). These toxicity measurements showed that variants containing the substitutions I408E, M755K, and Y776N displayed higher LC₅₀ values than Vip3Aa64. Preliminary mortality assays using lysates containing Vip3A-TR1 or Vip3Aa64(I408E/M755K) indicated that the reduced toxicity of some variants was primarily associated with the presence of Y776N (Table S03). Consequently, a new variant, Vip3A-TR6, was generated by removing this substitution. Vip3A-TR6 exhibited a 5.1 °C increase in *T*m relative to Vip3Aa64 without compromising insecticidal activity. Compared with Vip3A-TR5, the *T*m of Vip3A-TR6 decreased by 1.4 °C, consistent with the thermal stability differences observed between Vip3Aa64(DV) and Vip3Aa64(DV(Y776N)).

Residue Y776 is located in the highly exposed loop region 770–778 of domain V. The observed reduction in toxicity upon its substitution, together with its structural exposure, suggests that Y776 might contribute to the function of domain V. Although the precise function of domain V remains unclear, it likely interacts with chitinous carbohydrates in the larval peritrophic membrane^24^^55^,. In contrast, residues I408 and M755 are located at the interface with domain III and are unlikely to have a functional role in insecticidal activity, which explains why their substitution does not impact toxicity. Similarly, the function of domain IV remains poorly understood and is presumed to involve carbohydrate binding^55^, yet no binding to the peritrophic membrane was observed^24^. Previous studies have shown that domains IV and V do not bind to the midgut epithelium of *S. frugiperda*^24^.

#### Prolonged heat treatment and storage experiments of Vip3A-TR6

**Fig. 4:**
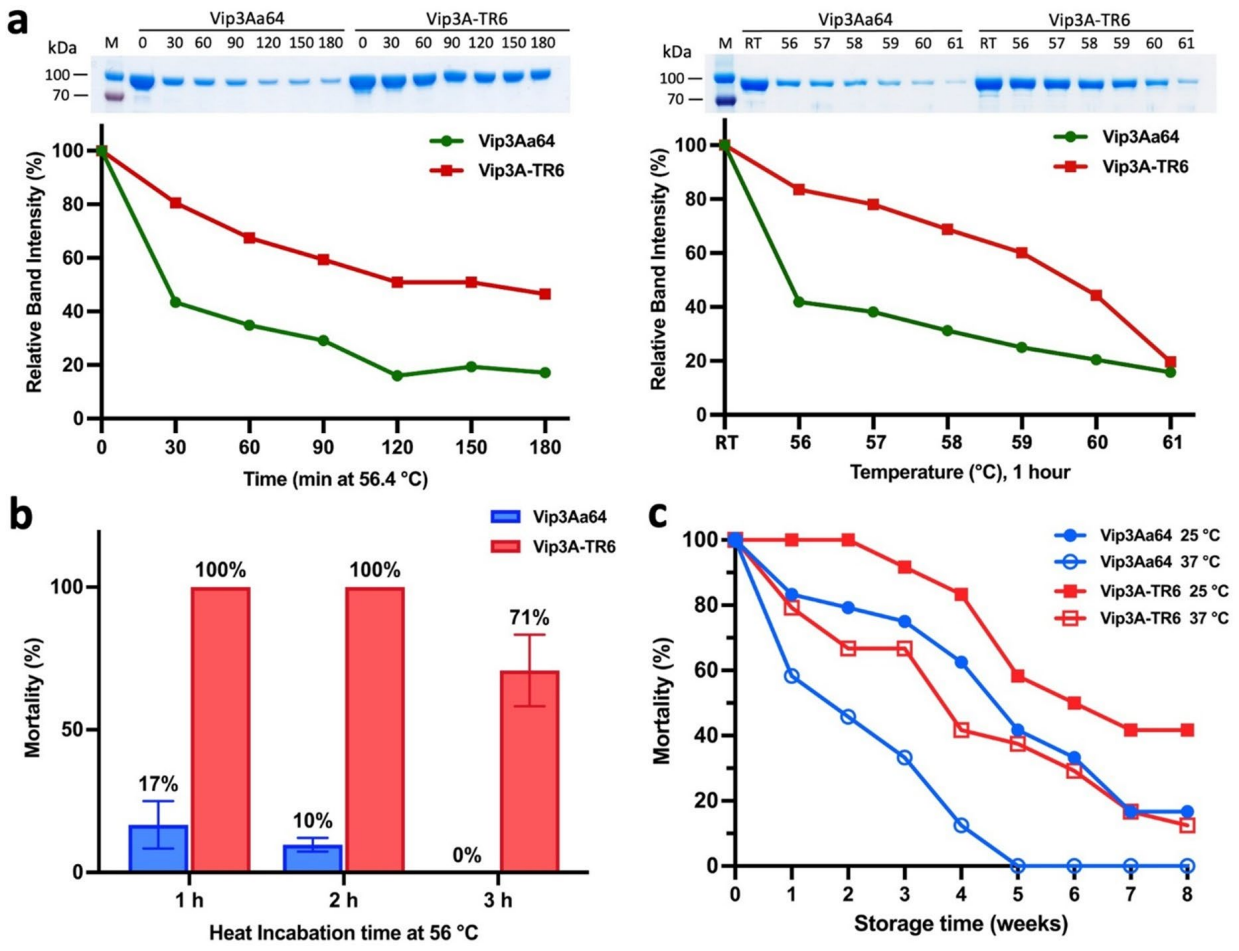
Heat-resistance of Vip3Aa64 and Vip3A-TR6. (**a**) Heat-induced precipitation analysis. SDS-PAGE gel showing the soluble protein fractions of Vip3Aa64 and Vip3A-TR6 (0.5 mg/mL) after incubation at RT (control), and at 55–61 °C for 1 h (a) and at 56.4 °C (*T*m value of Vip3Aa64) for 3 h (sample taken every 30 min; see Fig. S08 for uncropped image). Relative band intensities were quantified using ImageJ and normalized to the band intensity of 0-min sample. (**b**) Insecticidal activity after heat treatment. The purified wild-type Vip3Aa64 and Vip3A-TR6 (2 µg/cm²) were incubated at 56 °C (*T*m of Vip3A WT) for different time points (1–3 h). (**c**) The residual insecticidal activity of *Vip3A and Vip3A-TR5* during storage at 25 and 37 °C. The incubated proteins were tested with *S. exigua* weekly over a two-month storage period at 25 and 37 °C at a concentration of 2 µg/cm^2^. Mortality was monitored for seven days. Experiments were conducted as a single assay (no replicates), using 32 larvae per time point.

Vip3A-TR6 displayed a 5.1 °C increase in *T*m relative to Vip3Aa64 in NanoDSF measurements and exhibited equivalent insecticidal activity. However, the impact of this enhanced thermal resistance on long-term performance required further evaluation. To assess this, Vip3A-TR6 and Vip3Aa64 were incubated for 1 h at temperatures between 56 °C and 61 °C, and for up to 3 h at 56.4 °C with samples taken every 30 min. After centrifugation to remove aggregated material, the remaining soluble protein was estimated by band intensity on SDS-PAGE gels (see Fig. 4a). Residual insecticidal activity against *S. exigua* was measured following incubation at 56 °C for up to 3 h and after storage at 25 °C and 37 °C for up to 8 weeks (Fig. 4b and **c**).

The relative amounts of soluble Vip3A-TR6 and Vip3Aa64 estimated from SDS-PAGE band intensities were consistent with the *T*m values determined by NanoDSF. After 1 h at 56.4 °C, most Vip3Aa64 (*T*m: 56 °C) had aggregated, whereas more than half of the Vip3A-TR6 (*T*m: 61.5 °C) remained soluble. Similar to Vip3Aa64 incubated at 56.4 °C, Vip3A-TR6 largely aggregated after incubation at 61 °C, a temperature close to its *T*m. Because some fraction of denatured protein might remain soluble (e.g., Vip3Aa64 band intensity remained at ~ 20% after 3 h at 56.4 °C and after 1 h at 61 °C) and because in-gel quantification has limited precision, these values should be interpreted as estimates for the amount of denatured protein. Nonetheless, the results clearly demonstrate that Vip3A-TR6 has increased resistance to heat-induced aggregation, confirming its enhanced thermal resistance.

The improved thermal resistance of Vip3A-TR6 was further reflected in the residual toxicity after heat incubation and long-term storage (Fig. 4b, c). Vip3A-TR6 retained 100% activity after 1 and 2 h of incubation at 56 °C, whereas the toxicity of Vip3Aa64 declined to approximately 17% and 10%, respectively. After 3 h, Vip3A-TR6 activity decreased to 71%, whereas Vip3Aa64 lost all detectable activity. The aggregation assays (Fig. 4a) showed that Vip3A-TR6 denatures after prolonged incubation at 56.4 °C, suggesting that an overdose was used in the toxicity assays. Given this likely protein excess, the 71% mortality observed for Vip3A-TR6 after 3 h at 56 °C is consistent with the ~ 50% aggregation estimated from the band intensity. The complete loss of activity for Vip3Aa64 after 3 h at 56 °C, despite a small amount of soluble protein remaining (Fig. 4a), which likely reflects that a fraction of denatured protein remains soluble.

The long-term storage experiments showed the same trend as observed in the aggregation and toxicity assay after heat incubation, Vip3A-TR6 samples retain more toxicity compared to Vip3Aa64 samples under all tested conditions. At storage at 25 °C, Vip3Aa64 samples maintained high activity for the first two weeks but gradually declined, resulting in 16.7% activity by week eight. In contrast, Vip3A-TR6 samples stored at 25 °C, retained nearly complete activity for the first three weeks and still exhibited 41.7% toxicity after eight weeks. Vip3Aa64 samples stored at 37 °C lost activity quickly, dropping to 58.3% in one week and becoming completely inactive by week five. Vip3A-TR6 samples maintained about 37.5% activity by week five and 12.5% by week eight.

#### Production of Vip3A-TR6 in *B. thuringiensis*

The production of Vip3A toxins is typically carried out in *B. thuringiensis*, as the protein is secreted into the culture medium and therefore no cell disruption is required. However, *B. thuringiensis* also secretes other proteins, including proteases, which could reduce the benefit of improved thermal resistance, introduced substitutions could also have increased the susceptibility to proteolytic degradation. To evaluate these possibilities and to confirm that Vip3A-TR6 is secreted in the same manner as Vip3Aa64, Vip3A-TR6 was expressed in the natural producer strain *B. thuringiensis* 294 (Bt294).

Bt294 was modified using CRISPR-Cas9-mediated genome editing to generate the strain Bt294(Vip3A-TR6). Vip3A-TR6 was secreted into the culture supernatant at levels comparable to Vip3Aa64, indicating that the introduced substitutions do not impair secretion or promote degradation. To assess thermal resistance in the context of the culture medium, supernatants from Bt294 and Bt294(Vip3A-TR6) were incubated at 50, 55, 60, and 65 °C for 30 min prior to toxicity testing against *S. exigua* (see Table 2).

**Table 2:** Larvicidal activity of Bt294 and Bt294(Vip3A-TR6) supernatants after heat treatment at different temperatures and prolonged incubation at 55 °C. Bioassays were conducted against *S. exigua* larvae at 0.5 µg cm⁻². Values represent mean ± standard deviation from three independent colonies.

Condition	Mortality (%)	
	Bt294	Bt294(Vip3A-TR6)
Control (no heat treatment)	100 ± 0	100 ± 0
50 °C, 30 min	100 ± 0	100 ± 0
55 °C, 30 min	56 ± 26	100 ± 0
55 °C, 60 min	26 ± 13	100 ± 0
55 °C, 120 min	17 ± 4	100 ± 0
60 °C, 30 min	31 ± 10	25 ± 22
65 °C, 30 min	7 ± 2	3 ± 2

Up to 55 °C and 120 min of incubation, Vip3A-TR6 retained full insecticidal activity, consistent with the results obtained using purified protein. In contrast, Vip3Aa64 began to lose activity after only 30 min at 55 °C, which also aligns well with the purified-protein data. After incubation at 60 °C, the supernatants of both Bt294 and Bt294(Vip3A-TR6) displayed similar levels of residual toxicity. This outcome was unexpected, as Vip3Aa64 should be largely denatured at this temperature; however, the reduction in Vip3A-TR6 activity is consistent with the aggregation measurements (see Fig. 4a), which showed that approximately 50% of the protein becomes insoluble after 1 h at 60 °C. Following 30 min at 65 °C, nearly all activity was lost in the samples of both proteins, as anticipated for a temperature exceeding the *T*m of both Vip3A variants.

SDS-PAGE analysis of culture supernatants incubated for 30 min at the three temperatures (see Fig. S09) support the toxicity results. The band for full-length Vip3A-TR6 remained detectable up to 60 °C, while the Vip3Aa64 band was nearly undetectable at 55 °C. Overall, the performance of Vip3A-TR6 in culture supernatant closely matched the results obtained with purified protein, demonstrating that Vip3A-TR6 outperforms Vip3Aa64 under both conditions. Minor discrepancies between the two experimental setups are likely attributable to differences between the culture medium and buffer systems. In addition, the presence of proteases in the culture supernatant may contribute to toxin degradation; SDS-PAGE analysis of Vip3A-TR6 after 30 min at 60 °C revealed new, lower-molecular-weight bands indicative of proteolysis (Fig. S09).

## Conclusion

In this study, we engineered a thermally stabilized Vip3Aa64 variant, Vip3A-TR6 (Vip3Aa64(I408E/M755K/N633V/G580E)), which exhibits a 5.1 °C increase in *T*m while maintaining insecticidal activity comparable to that of the wild-type protein. Vip3A-TR6 can be produced in the native host *B. thuringiensis* and, like Vip3Aa64, is efficiently secreted into the culture medium, indicating that the introduced substitutions do not impair expression, secretion, or in vivo stability.

Vip3A-TR6 was generated using a combined rational design and random mutagenesis strategy targeting the thermally labile domains IV and V. NanoDSF screening performed directly in clarified lysates using intrinsic tryptophan fluorescence enabled rapid identification of stabilizing mutations and provides a broadly applicable workflow for protein engineering. Structural considerations suggest that the stabilizing substitutions enhance interdomain interactions and reinforce local packing within domain IV.

Thermal incubation and long-term storage experiments with both purified protein and *B. thuringiensis* culture supernatants demonstrated that Vip3A-TR6 exhibits markedly enhanced resistance to heat-induced aggregation and retains biological activity under conditions that rapidly inactivate Vip3Aa64. These results confirm that the *T*m is a reliable predictor of functional stability and highlight the value of protein engineering for improving the robustness of Vip3A toxins.

Collectively, these findings establish Vip3A-TR6 as a thermally resilient and biologically potent Vip3A variant with clear potential to enhance the durability and field performance of Vip3A-based biopesticides. This work provides a framework for the rational improvement of Vip toxins and contributes to the development of more stable and reliable biological control agents for lepidopteran pest management.

## Supplementary Information

Below is the link to the electronic supplementary material.

Supplementary Material 1

## Data Availability

The raw data supporting the findings of this study are available from the corresponding author upon reasonable request.
