# Supplementary material for: Domain-guided engineering of a thermoresistant Vip3A toxin for enhanced functional robustness
Source: Sci Rep. 2026 Apr 21;16:13016. doi: 10.1038/s41598-026-47865-0 (PMC13099987; doi:10.1038/s41598-026-47865-0)
Supplement: Supplementary file 1 — Supplementary Material 1 [file 41598_2026_47865_MOESM1_ESM.docx]

**Supporting Information**

**Domain-Guided Engineering of a Thermoresistant Vip3A Toxin for Enhanced Functional Robustness**

Thanapon Kunlawatwimon^1,2^, Florian Bourdeaux^2,3*^, Panadda Boonserm^1^, Sumarin Soonsanga^4^, Julian Luka^3^, Boonhiang Promdonkoy^4^, Ulrich Schwaneberg^2,3*^

^1^ Institute of Molecular Biosciences, Mahidol University, Salaya, Phuttamonthon, Nakhon Pathom 73170, Thailand

^2^ DWI - Leibniz-Institute for Interactive Materials, Aachen 52074, Germany

^3^ Institute of Biotechnology, RWTH Aachen University, 52074 Aachen, Germany

^4^ National Center for Genetic Engineering and Biotechnology, National Science and Technology Development Agency, 113 Pahonyothin Road, Khlong Nueng, Khlong Luang, Pathum Thani 12120, Thailand

*E-mail: [bourdeaux@dwi.rwth-aachen.de](mailto:bourdeaux@dwi.rwth-aachen.de)

*E-mail: [u.schwaneberg@biotec.rwth-aachen.de](mailto:u.schwaneberg@biotec.rwth-aachen.de)

**Table S01:** Primers used to construct the main Vip3Aa64 variants. For and Rev stand for forward and reverse primers, respectively. Underlines indicate mutagenic sites, and Tm is the predicted melting temperature of the primers.

| Primer’s name | Sequence (5′-3′) | *T*_m_ (˚C) |
| --- | --- | --- |
| I408E-For | GCCCAGATCAATCTGAACAAGAATATTATACAAATAACATAGTATTTCCAAATGAATATG | 62.8 |
| I408E-Rev | TTGTTCAGATTGATCTGGGCACAA | 56.9 |
| M755K-For | TTCACTACAAAATTTGAGAAAGATAACTTTTATATAG | 54.0 |
| M755K-Rev | TTCTCAAATTTTGTAGTGAATTTTTCAGAAACATCTTTAGCACCGCT | 64.1 |
| Y776N-For | TTTCTCAAGGGAATAATTTAAACGGTGGTCCTATTGTACATTTTTACGA | 63.9 |
| Y776N-Rev | TAAATTATTCCCTTGAGAAAGCTCTATATAAAA | 53.6 |
| K557P-For | AGCCGTGGAAAGCAAATAATCCGAATGCGTATGTAGATCATACAGGCG | 69.4 |
| K557P-Rev | ATTATTTGCTTTCCACGGCTCTAAATTGTCCTCTT | 61.9 |
| K557E-For | AGCCGTGGAAAGCAAATAATGAGAATGCGTATG  TAGATCATACAGGCG | 68.3 |
| K557E-Rev | ATTATTTGCTTTCCACGGCTCTAAATTGTCCTCTT | 61.9 |
| G580R-For | TATATGTTCATAAGGACGGAAGAATTTCACAATTTATTGGAGATAAGTTAAAACC | 62.7 |
| G580R-Rev | TCCGTCCTTATGAACATATAAAGCTTTAGTTCCAT | 59.5 |
| G580E-For | TATATGTTCATAAGGACGGAGAAATTTCACAATTTATTGGAGATAAGTTAAAACC | 62.7 |
| G580E-Rev | TCCGTCCTTATGAACATATAAAGCTTTAGTTCCAT | 59.5 |
| N633S-For | TAGAAGATTATCAAACTATTAGTAAACGTTTTACTACAGGAACTGATTTAAAGG | 61.4 |
| N633S-Rev | AATAGTTTGATAATCTTCTAAATTATTATTTGTAT | 50.3 |
| N633V-For | TAGAAGATTATCAAACTATTGTGAAACGTTTTACTACAGGAACTGATTTAAAGG | 62.7 |
| N633V-Rev | AATAGTTTGATAATCTTCTAAATTATTATTTGTAT | 50.3 |

**Table S02:** List of substitutions done in domain V and the corresponding melting point determined in lysate via nanoDSF. Cells were lysed via sonication and lysate clarified by centrifugation. DV: Vip3Aa64(DV). Ox: lysate kept open for about 30 min. Red.: DTT added to the lysate (final concentration 10 mM). Average and standard deviation of three technical replicates. DV(L769W) no melting point was determined, since the protein was not obtained in soluble form (inclusion bodies were formed).

| Variant | *T*m (°C) |
| --- | --- |
| DV | 47.9 ± 0.0 |
| DV(E738K) | 45.9 ± 0.0 |
| DV(E761K) | 44.2 ± 0.0 |
| DV(V786F) | 46.0 ± 0.0 |
| DV(Q709L) | 36.9 ± 0.1 |
| DV(F760Y) | 48.0 ± 0.1 |
| DV(L769W) | - |
| Vip3Aa35(DV) | 48.9 ± 0.1 |
| DV(M755I) | 48.1 ± 0.0 |
| DV(F760L) | 48.1 ± 0.0 |
| DV(F761G) | 45.4 ± 0.1 |
| DV(Y776N) | 50.5 ± 0.0 |
| DV(H782K) | 48.2 ± 0.0 |
| DV(Y784S) | 45.6 ± 0.1 |
| **Cyclized variants** | ***T*m (°C)** |
| DV(Y776N)  (control) | Ox. 50.7 ± 0.1  Red. 50.6 ± 0.1 |
| DV(S765C/K789C) | Ox. 55.1 ± 0.1  Red. 45.1 ± 0.0 |
| DV(S765C)-C | Ox. 60.9 ± 0.1  Red. 45.7 ± 0.0 |
| DV(S765C)-GC | Ox. 64.8 ± 0.2  Red. 52.2 ± 0.1 |
| DV(S765C)-GGC | Ox. 63.3 ± 0.1  Red. 50.8 ± 0.1 |
| DV(S765C/K789G)-C | Ox. 56.8 ± 0.1  Red. 41.8 ± 0.0 |
| Vip3Aa64(S675C)-C | Ox. 56.6 ± 0.0  Red. 47.5 ± 0.1 |
| Vip3Aa64(S675C)-GGC | Ox. 55.9 ± 0.0  Red. 51.9 ± 0.0 |

**Table S03:** Mortality rates from preliminary insect bioassays. These experiments were conducted prior to the more detailed characterization studies. Overnight cultures (5 mL TB) of *E. coli* BL21 expressing the indicated proteins were harvested by centrifugation and resuspended in 2.5 mL water. Aliquots of 50 µL of the resulting cell suspension were applied onto the surface of an artificial diet in 24-well plates. For each construct, 12 larvae (one per well) were tested, and mortality was assessed after 7 days. Any reduction in mortality reflects a substantial loss of activity, as the applied toxin dose exceeded the minimum required for full efficacy.

| Constructs | Mortality (%) |
| --- | --- |
| Vip3Aa64 | 100 |
| eGFP-Vip3Aa64* | 100 |
| Vip3Aa64-eGFP* | 0 |
| Vip3Aa64(K557E) | 100 |
| Vip3Aa64(G580E) | 100 |
| Vip3Aa64(N633V) | 100 |
| Vip3Aa64(I408E/M755K) | 100 |
| Vip3Aa64(I408E/M755K/Y776N) | 75 |
| Vip3Aa64(I408E/M755K/Y776N/S675C/K789C)** | 33.3 |

*: eGFP fusions of Vip3Aa64 were linked via a 17 amino acid long helix linker (AEAAAKEAAAKEAAAKA).

**: Addition of the cyclized domain to the Variant Vip3Aa64(I408E/M755K/Y776N) caused a significant drop in toxicity indicating the detrimental effects of the modified domain V.

**Table S04:** Identified Vip3Aa64(DI-IV) variants with improved *T*m. NanoDSF measurements of purified proteins. V5 and V6 are based on FoldX, I-Mutant, and DeepDDG (see **Table S05**) predictions. Predicted substitutions were introduced into Vip3Aa64(DI-IV).

| Variant | Substitution | *T*m (°C) |
| --- | --- | --- |
| Vip3Aa64 | - | 56.4 |
| Vip3Aa64(DI-IV) | - | 58.1 |
| V1 | K557E | 61.1 |
| V2 | G580R | 63.4 |
| V3 | G580E | 64.7 |
| V4 | N633S | 61.1 |
| **Predicted** |  |  |
| V5 | K557P | 59.9 |
| V6 | N633V | 62.9 |

**Table S05:** *T*m values of single point substitutions in domain IV introduced into Vip3Aa64 (full length). Measurement was performed in clarified lysate with only one replicate.

| **Vip3Aa64** | ***T*m (°C)** |
| --- | --- |
| Vip3Aa64 | 56.7 |
| G580E | 58.6 |
| K557E | 57.4 |
| N633V | 58.1 |

**Table S06:** Predicted substitutions in domain IV by FoldX, I-Mutant, and DeepDDG.

| Residue | Substitutions | Prediction |
| --- | --- | --- |
| 537 | N537D | Loop between D3-D4 |
| 557 | K557P | Increase rigidity |
| 570 | T570H | Salt-bridge with D660 |
| 582 | S582L | Increase hydrophobic --> Beta-sheet |
| 583 | Q583I | Increase hydrophobic |
| 611 | D611P | Increase rigidity |
| 618 | H618M | Increase rigidity |
| 633 | N633V | Increase hydrophobic |
| 641 | D641S | H-bond with N613 |
| 660 | D660I | Increase hydrophobic |


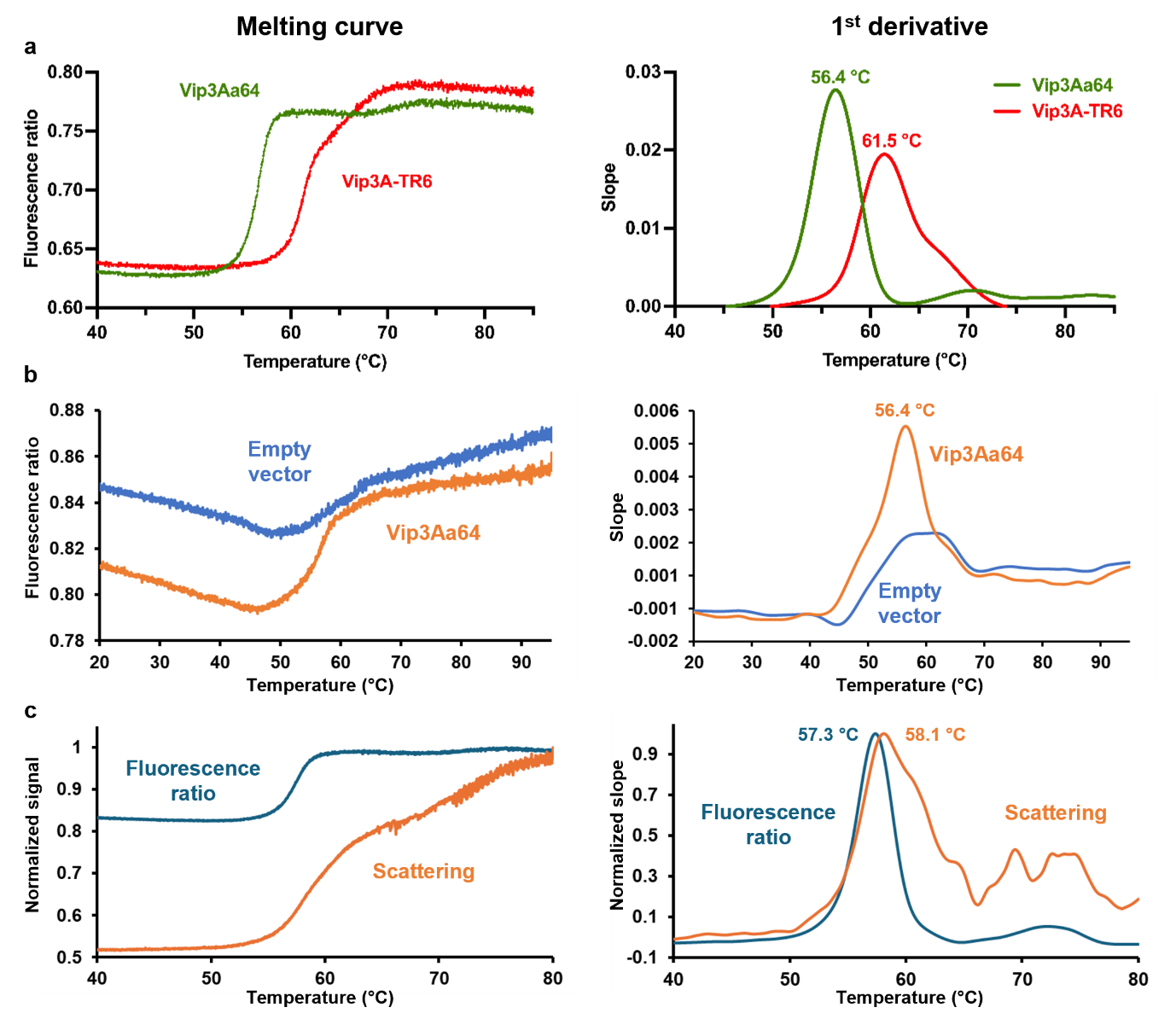


**Figure S01**. NanoDSF measurements with purified Vip3A toxins and Vip3Aa64 in lysate. (a) NanoDSF measurements of purified Vip3A toxins. (b) NanoDSF measurements of Vip3Aa64 in lysate and an empty vector control (lysate). *E. coli* proteins contained in the lysate create a broad background signal from about 45 °C to 70 °C, while Vip3Aa64 has a more distinct peak. Lysate measurements are limited by the ratio of protein-of-interest to *E. coli* proteins, basically the method is limited to well expressed and soluble proteins, or to proteins with melting points outside of 45 °C to 70 °C. (c) Normalized fluorescence ratio and scattering data for Vip3Aa64. Measurements were performed using approximately 1.5 mg/mL purified Vip3Aa64 in buffer (200 mM NaCl, 50 mM Tris, pH 8.0, and 8.6% glycerol). Reliable scattering measurements required protein concentrations above 1 mg/mL (for Vip3Aa64 and its variants), as lower concentrations resulted in insufficient signal intensity. Additionally, low ionic strength further reduced the scattering signal. Typical background signals ranged from 80-85 mAU (buffer-filled capillary), while signals after thermal denaturation increased to 150-200 mAU (here, 162 mAU), depending on protein concentration. The observed increase in melting temperature is likely attributable to the higher protein concentration used in this experiment, as other samples were measured at or below 0.5 mg/mL.


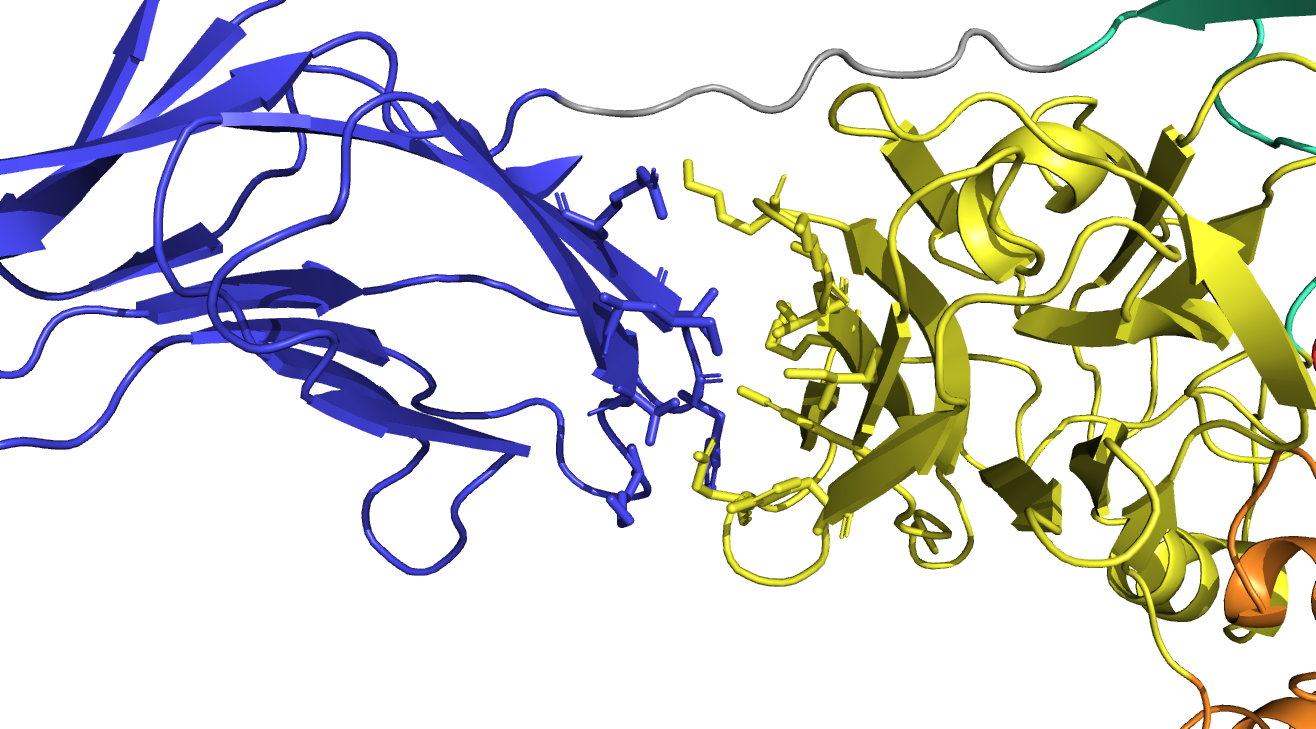


**Figure S02**. Cartoon depiction of the domain III and domain V interface. Vip3Aa64 (Swiss-Model based on PDB-ID: 6tfj). Domain III: yellow. Domain IV: green-cyan. Domain V: blue. Linker: gray. Residues involved in the interface are shown as sticks.


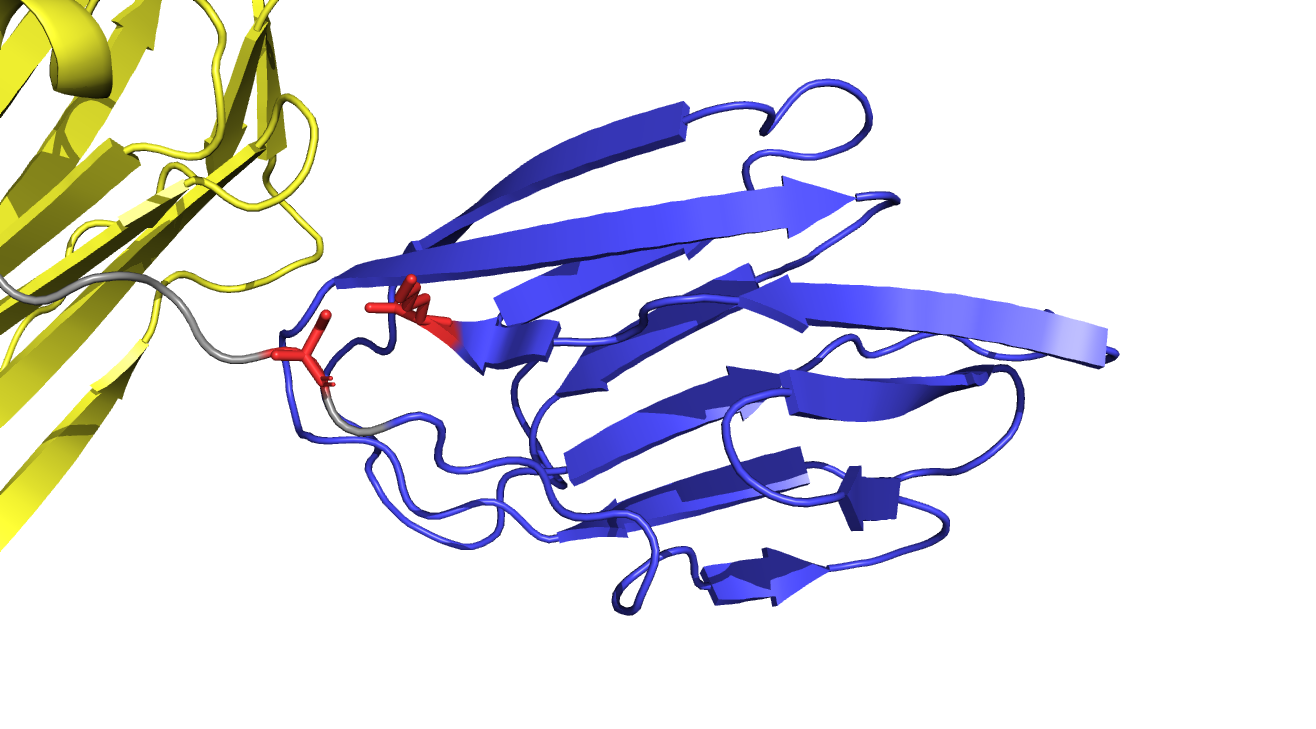


**Figure S03**. Cartoon depiction of domain V. Vip3Aa64 (Swiss-Model based on PDB-ID: 6tfj). Domain V: blue. Linker: gray. Domain III: yellow. Residues S765 and K789: red.


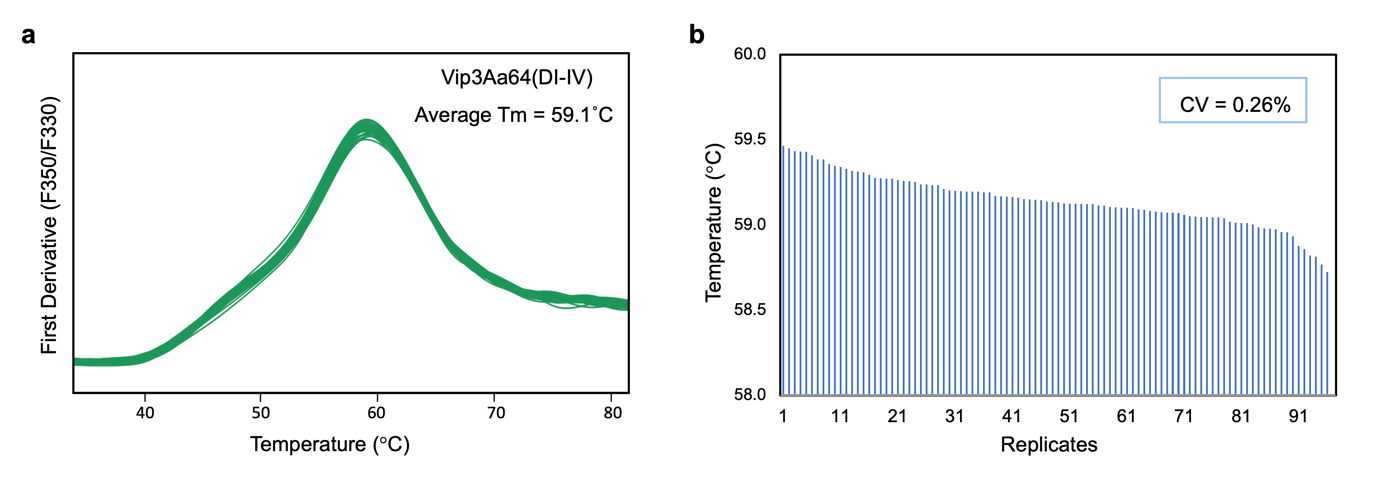


**Figure S04.** Validation of the thermal resistance screening with Vip3Aa64(DI-DiV) variants in lysate using NanoDSF. (a) Thermal unfolding profiles from NanoDSF, showing the fluorescence intensity ratio changes as a function of temperature for all 96 replicates with an average Tm of 59.1 °C. (b) Bar graph representation of melting temperatures (*T*m) across 96 replicates and a coefficient of variation (CV) of 0.26%, confirming the robustness and consistency of the screening method.

**Figure S05 is divided into multiple pages.**

**Methods of the MD Simulations are in the figure caption.**

**
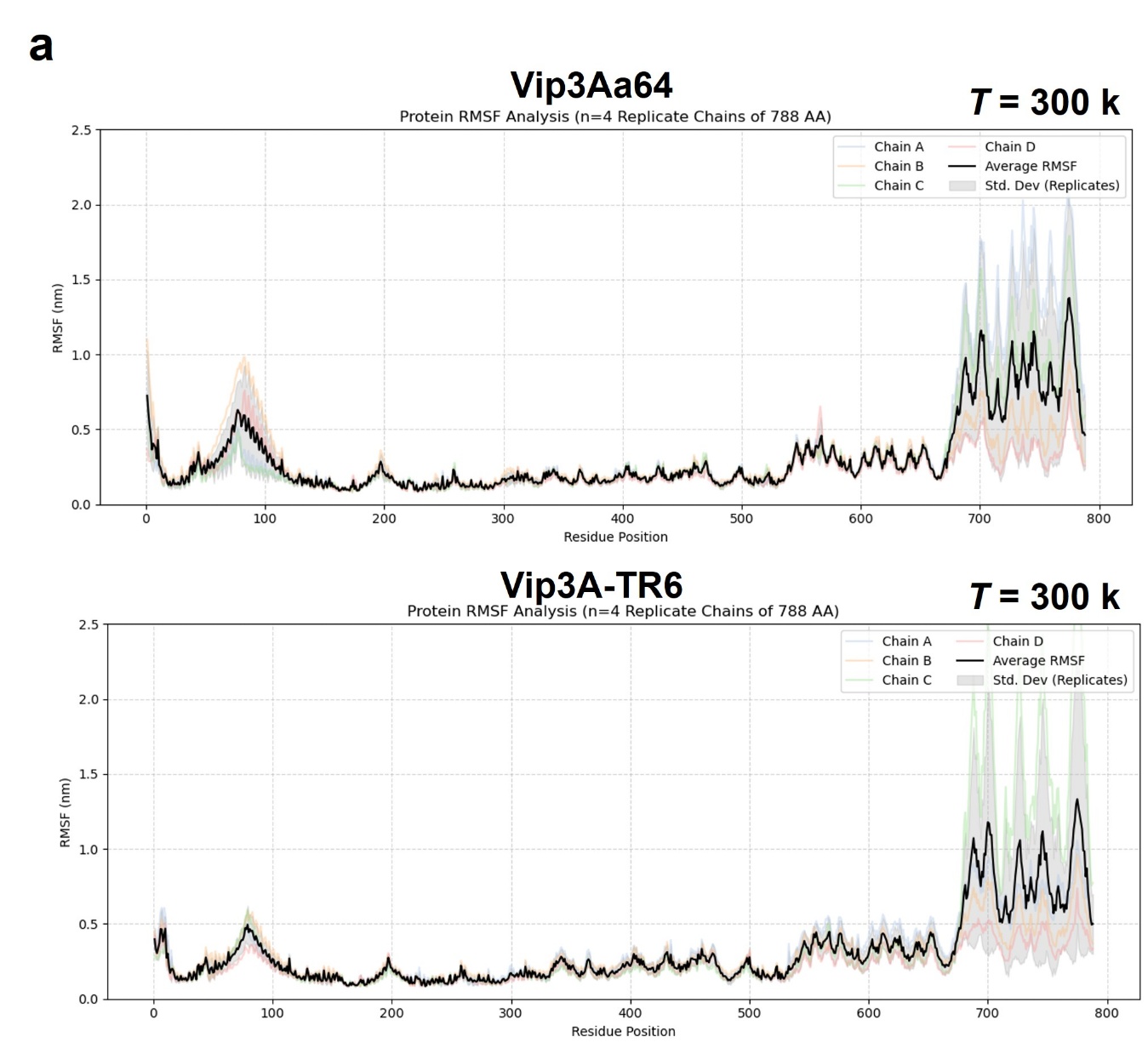
**


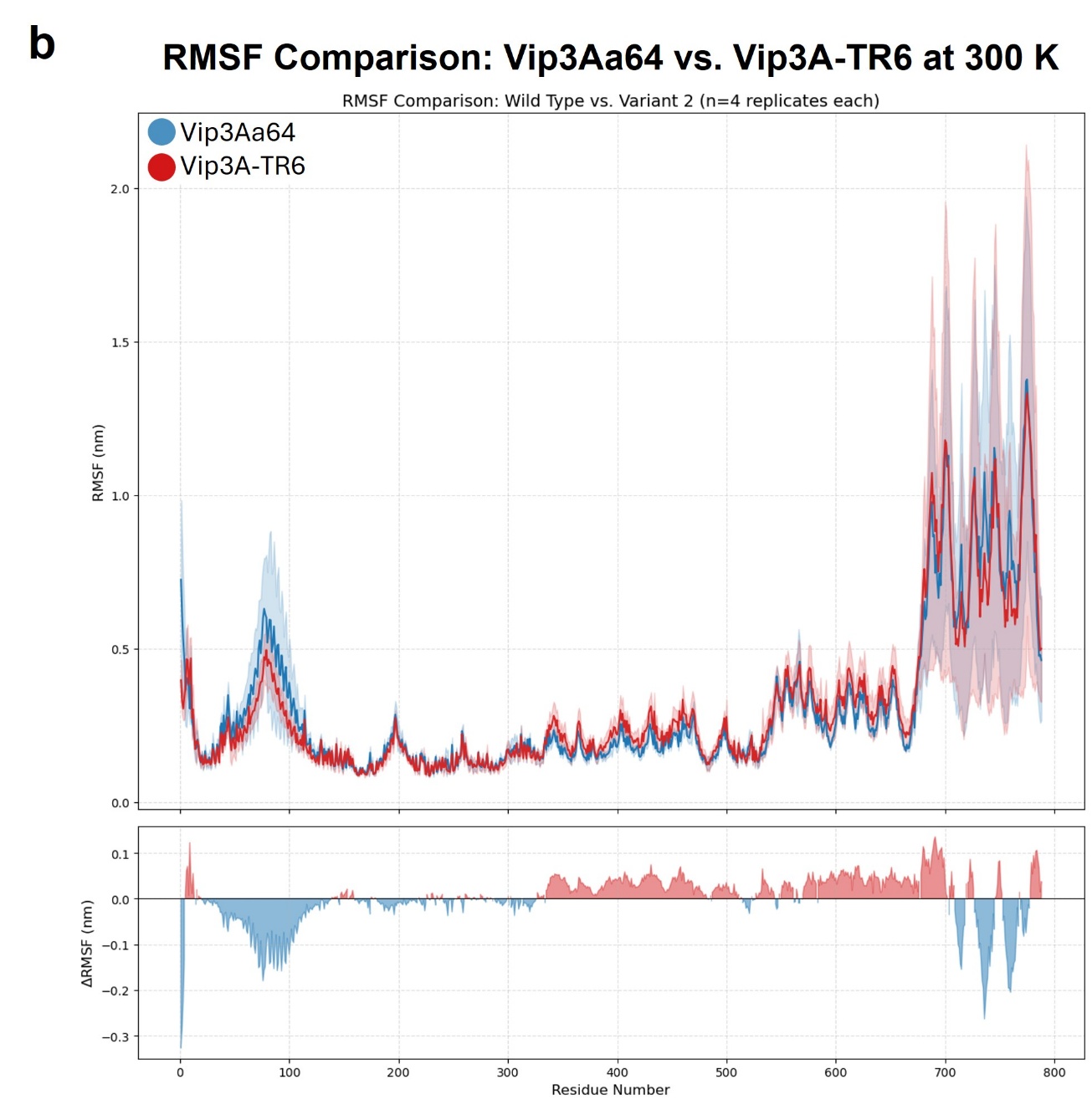


**
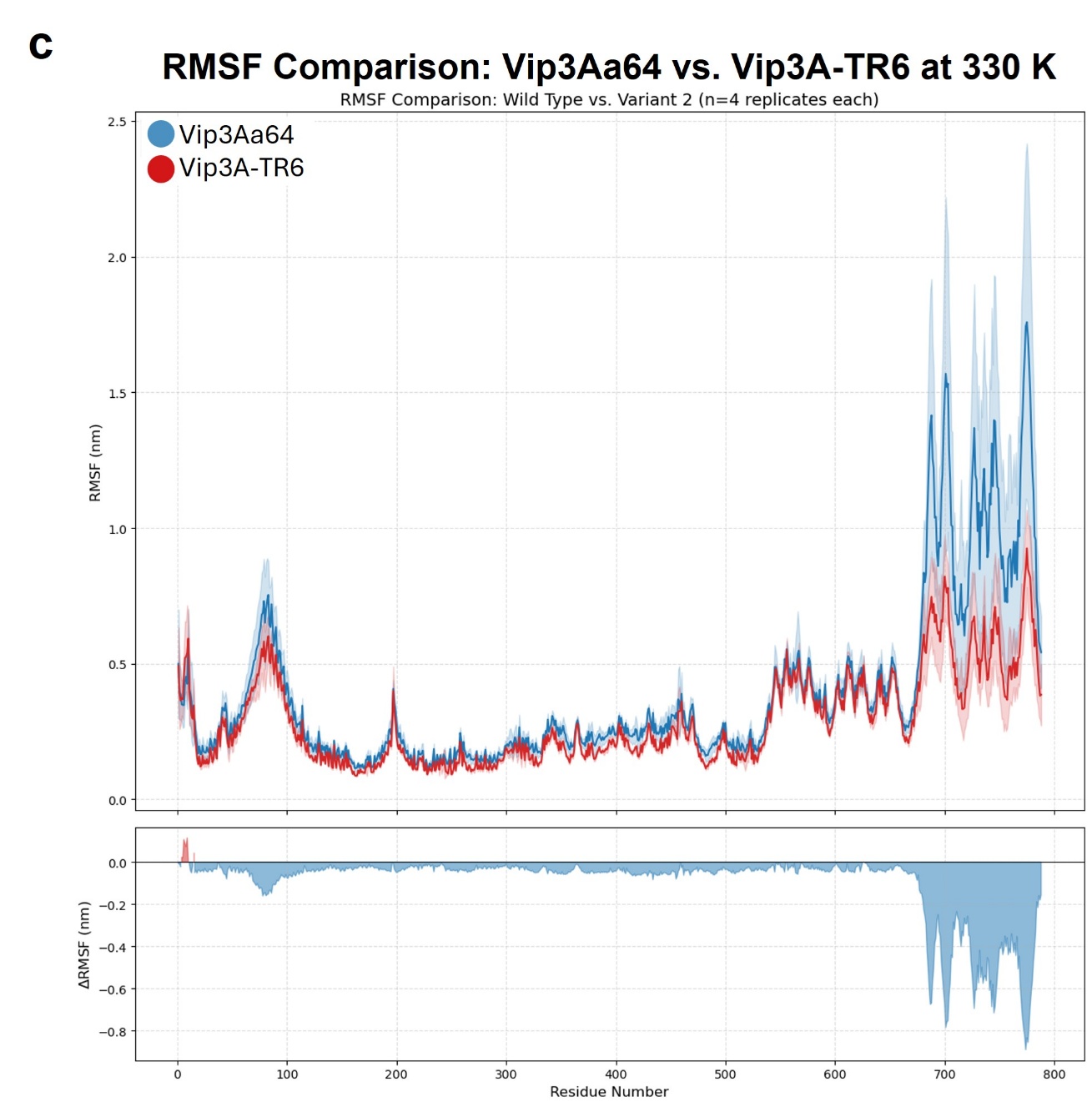
**
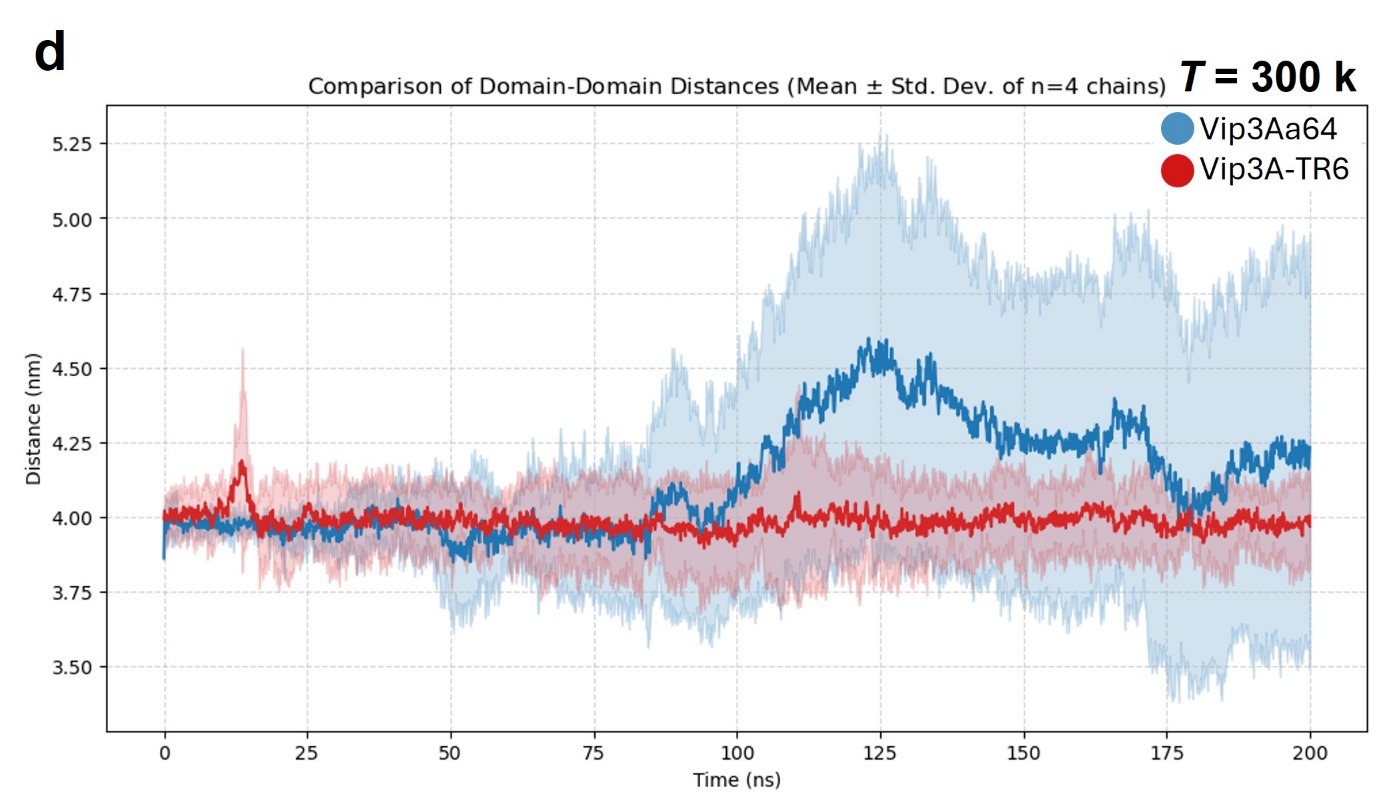


**Figure S05**. Results of the MD Simulations with Vip3Aa64 and Vip3A-TR6. (a) Root-mean-square fluctuation (RMSF) of side chains for Vip3Aa64 and Vip3A-TR6 at 300 K. (b) Comparison of side-chain RMSF values at 300 K. Domain V in Vip3A-TR6 exhibits reduced flexibility compared to Vip3Aa64. As domain I showed global rearrangements (transitioning from the C4 symmetry predicted by AlphaFold3 toward the C2 symmetry observed in PDB ID: 6TFJ), changes in this domain were excluded from further analysis. (c) Comparison of side-chain RMSF values at 330 K. The difference in flexibility of domain V between Vip3A-TR6 and Vip3Aa64 is more pronounced at elevated temperature. In contrast, domain IV appeared more rigid than expected, and no clear effect of the substitutions was observed. (d) Distance between the centers of mass of domains III and V during the simulations. In Vip3A-TR6, domain V exhibits reduced mobility and remains closer to domain III compared to Vip3Aa64.

**Methods:** Simulations were performed using GROMACS version 2021.5. The Force field CHARMM36 was used and initial protein structures were obtained from AlphaFold 3. Protonation states under the experimental pH conditions were determined using PROPKA, and the protonation was adjusted during topology generation. The protein was placed in a triclinic box and solvated with TIP3P water. Sodium chloride ions were added to neutralize existing charges and to mimic the ionic strength of PBS. The system was energy minimized using the steepest descent method, and equilibration was achieved by running an NVT ensemble at 300 K for 1 ns using the V-rescale thermostat. This step was followed by an NPT ensemble at 1 bar for 100 ps using the Parrinello-Rahman barostat. The NPT output was used to start a production run at 300K or 330 K for 200ns. Periodic boundary conditions were removed from the resulting trajectories and analysis was performed using *gmx rmsf* and *gmx mindist* commands.


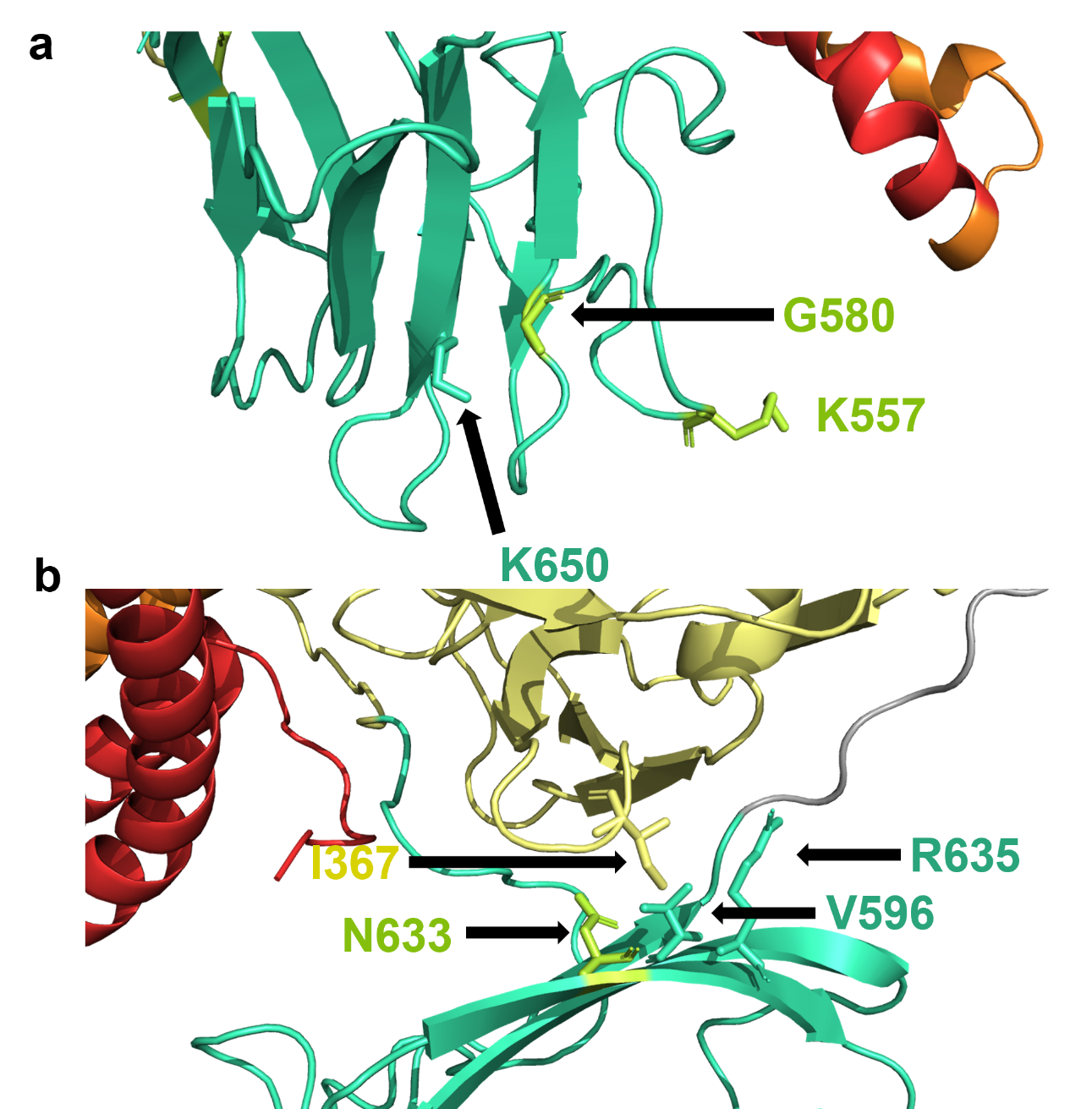


**Figure S06**. Cartoon depiction domain IV with certain residues depicted as sticks. (a) Location of the substitutions at G580 and K557 and potential interaction partners (shown in sticks). (b) Location of the substitutions at N633 and potential interaction partners (shown in sticks). Domain I: red. Domain II: orange. Domain III: yellow. Domain IV:green-cyan. Positions sunstituted in domain IV: yellow-green. Linker: grey.


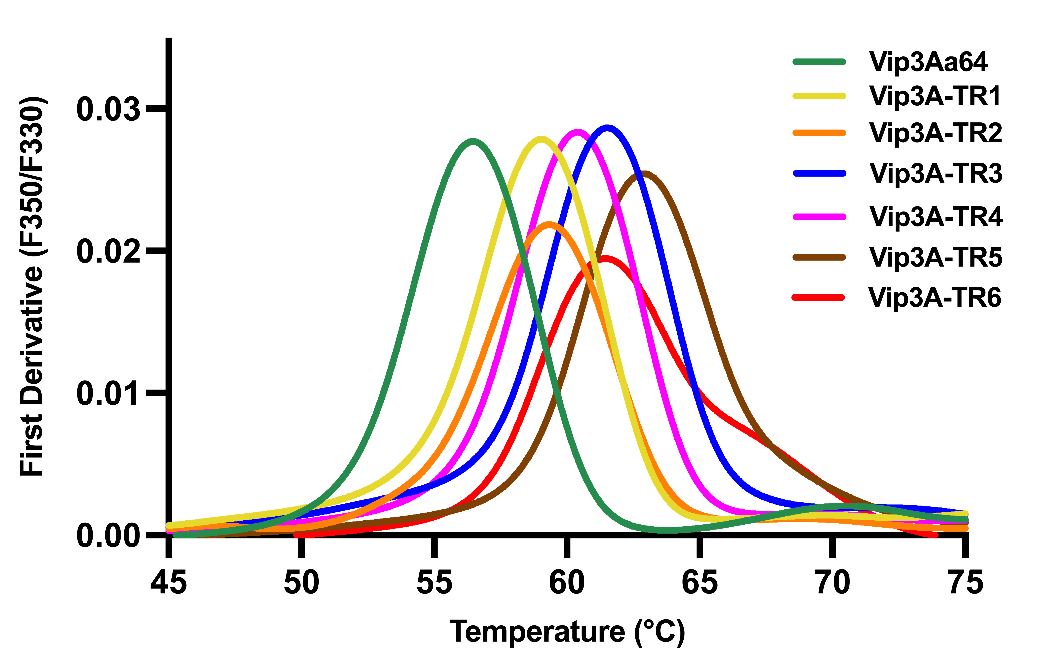


**Figure S07.** First derivative of the thermal unfolding profiles of the purified Vip3A-TR variants. The peaks correspond to the *T*m values. Vip3Aa64: green.

Vip3A-TR1 (Vip3Aa64(I408E/M755K/Y776N): yellow.

Vip3A-TR2 (Vip3Aa64(I408E/M755K/Y776N/K557E): orange.

Vip3A-TR3 (Vip3Aa64(I408E/M755K/Y776N/G580E): blue.

Vip3A-TR4 (Vip3Aa64(I408E/M755K/Y776N/N633V): pink.

Vip3A-TR5 (Vip3Aa64(I408E/M755K/Y776N/N633V/G580E): brown.

Vip3A-TR6 (Vip3Aa64(I408E/M755K/N633V/G580E): red.


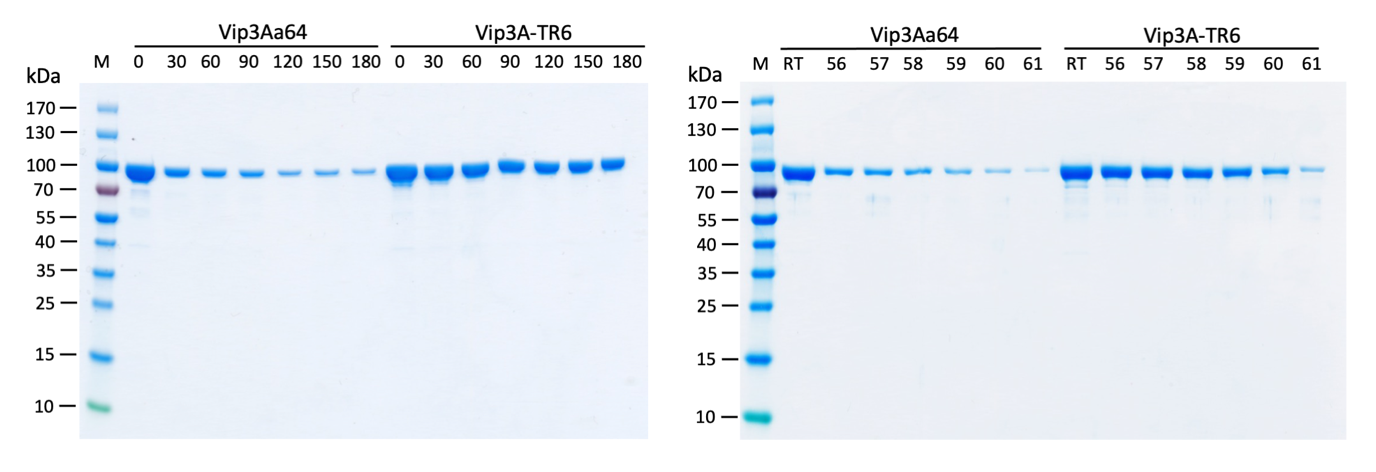


**Figure S07.** Uncropped SDS-PAGE gel of **Figure 04 a**.

**
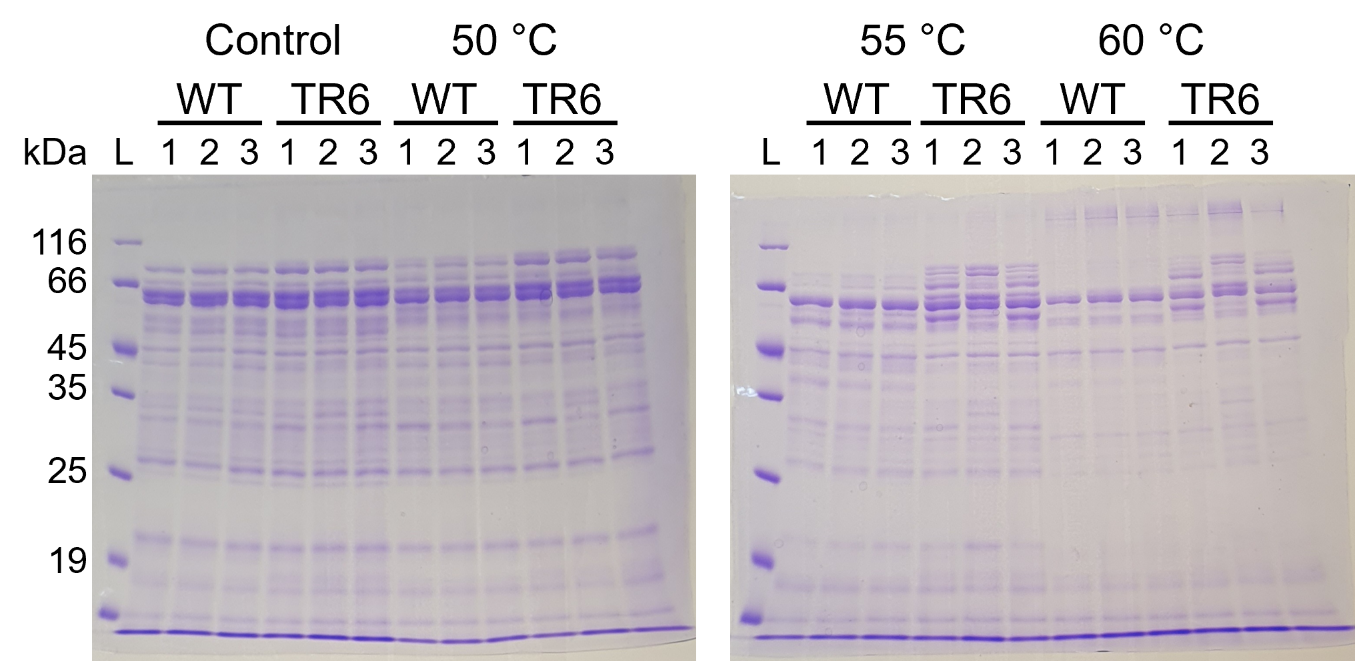
**

**Figure S09.** SDS-PAGE analysis of Bt294-WT and Bt294-Vip3A-TR6 supernatants after heat treatment. Culture supernatants were incubated at 50, 55, and 60 °C for 30 min before electrophoresis. The intensity of the full-length Vip3A band decreased progressively in Bt294-WT with increasing temperature, and was almost completely lost at 60 °C. In contrast, Vip3A-TR5 bands remained clearly visible at 50 and 55 °C, with faint but detectable intensity even at 60 °C. These results corroborate bioassay data, confirming that TR6 maintains structural integrity under moderate heat stress better than the wild type. Numbers represent individual colonies tested. L: ladder.
